# Network pharmacology and experimental verification based research into the effect and mechanism of Aucklandiae Radix–Amomi Fructus against gastric cancer

**DOI:** 10.1038/s41598-022-13223-z

**Published:** 2022-06-07

**Authors:** Siyuan Song, Jiayu Zhou, Ye Li, Jiatong Liu, Jingzhan Li, Peng Shu

**Affiliations:** 1 Affiliated Hospital of Nanjing University of Chinese Medicine, Nanjing, 210029 Jiangsu China; 2 Nanjing University of Chinese Medicine, Nanjing, 210029 Jiangsu China; 3 Jiangsu Provincial Hospital of Chinese Medicine, Nanjing, 210029 Jiangsu China

**Keywords:** Cancer therapy, Gastrointestinal cancer

## Abstract

To investigate the mechanism of the Aucklandiae Radix–Amomi Fructus (AR–AF) herb pair in treating gastric cancer (GC) by using network pharmacology and experimental verification. Using the traditional Chinese medicine system pharmacology database and analysis platform (TCMSP), the major active components and their corresponding targets were estimated and screened out. Using Cytoscape 3.7.2 software, a visual network was established using the active components of AR–AF and the targets of GC. Based on STRING online database, the protein interaction network of vital targets was built and analyzed. With the Database for Annotation, Visualization, and Integrated Discovery (DAVID) server, the gene ontology (GO) biological processes and the Kyoto Encyclopedia of Genes and Genomes (KEGG) signaling pathways of the target enrichment were performed. AutoDock Vina was used to perform molecular docking and calculate the binding affinity. The mRNA and protein expression levels of the hub targets were analyzed by the Oncomine, GEPIA, HPA databases and TIMER online tool, and the predicted targets were verified by qRT–PCR in vitro. Eremanthin, cynaropicrin, and aceteugenol were identified as vital active compounds, and AKT1, MAPK3, IL6, MAPK1, as well as EGFR were considered as the major targets. These targets exerted therapeutic effects on GC by regulating the cAMP signaling pathway, and PI3K-Akt signaling pathway. Molecular docking revealed that these active compounds and targets showed good binding interactions. The validation in different databases showed that most of the results were consistent with this paper. The experimental results confirmed that eremanthin could inhibit the proliferation of AGS by reducing the mRNA expression of hub targets. As predicted by network pharmacology and validated by the experimental results, AR–AF exerts antitumor effects through multiple components, targets, and pathways, thereby providing novel ideas and clues for the development of preparations and the treatment of GC.

## Introduction

There are approximately 400,000 new gastric cancer cases in China each year, including 350,000 deaths^1^. The incidence of gastric cancer ranks second among malignant tumors in China, and the mortality rate is third^2^. Although advances in surgical methods, radiotherapy, chemotherapy and neoadjuvant therapies have significantly improved the survival rate of GC patients, the outlook for patients with advanced GC remains disappointing due to the poor prognosis and limited treatment options^3^. Thus, traditional Chinese medicine, a complementary and alternative approach, is considered in the treatment of GC.

Recently, electronic technology has proven to be a more effective and rational method for developing drugs. The combination of network pharmacology and molecular docking is considered to be rapid, inexpensive, and effective in drug development^4^. Molecular docking and molecular dynamics use the three-dimensional (3D) structure of the target protein to study the specific structural features and interactions between the ligand and the protein. The main purpose of molecular docking is to reveal the main binding pattern of the ligand to the known 3D structure of the receptor. Therefore, this method can identify the correct position and orientation of the ligand in the protein binding pocket and predict the affinity between the ligand and protein^5^.

It has been verified that traditional Chinese medicine (TCM) has the characteristics of “multiple components, multiple targets, and multiple pathways” in the treatment of various diseases^6^. Studies have shown that TCM has advantages in improving the quality of life of GC patients^7^. Chronic atrophic gastritis is one of the precancerous lesions of GC, and Aucklandiae Radix–Amomi Fructus (AR–AF) is the main herb pair for the treatment of chronic atrophic gastritis^8–10^. AR invigorates the stomach, promoting qi and relieving pain. It is mainly used for the treatment of gastrointestinal diseases such as stagnation of qi in the spleen and stomach, diarrhea, tenesmus, poor food accumulation, vomiting and diarrhea^9^. Modern pharmacological research shows that AR has the effect of relieving smooth muscle spasms, lowering blood pressure, and exhibiting antibacterial activity. It also has certain anticancer, immune, and anti-inflammatory activities^11^. Terpenes, phenylpropanoids, lignans, flavonoids, and other compounds contained in the woody. It has a certain inhibitory effect on breast cancer^12^, liver cancer^13^, and prostate cancer^14^. Costanolactone induces apoptosis by activating the mitochondrial pathway in vitro and in vivo and inhibits the viability of BGC-823 cells in a concentration-dependent manner^15^. The IC50 values of cosanolide were 32.80 and 23.12 μM, respectively. AF has the functions of dispelling dampness and appetizing, warming the spleen and relieving diarrhea, regulating qi and relieving fetuses^10^. Modern pharmacological studies have shown that the main functional components include volatile substances such as bornyl acetate, camphor, limonene, and camphor, which can protect gastric mucosa, improve gastrointestinal function, relieve pain, relieve diarrhea, and promote the secretion of digestive juice^16^. The extract of flavonoids in AF at a concentration of 450 mg/kg has the best tumor suppressive effect and has an inhibitory effect on transplanted tumors S180 and H22, with a tumor suppression rate of 54.40%^17^.

**Figure 1:**
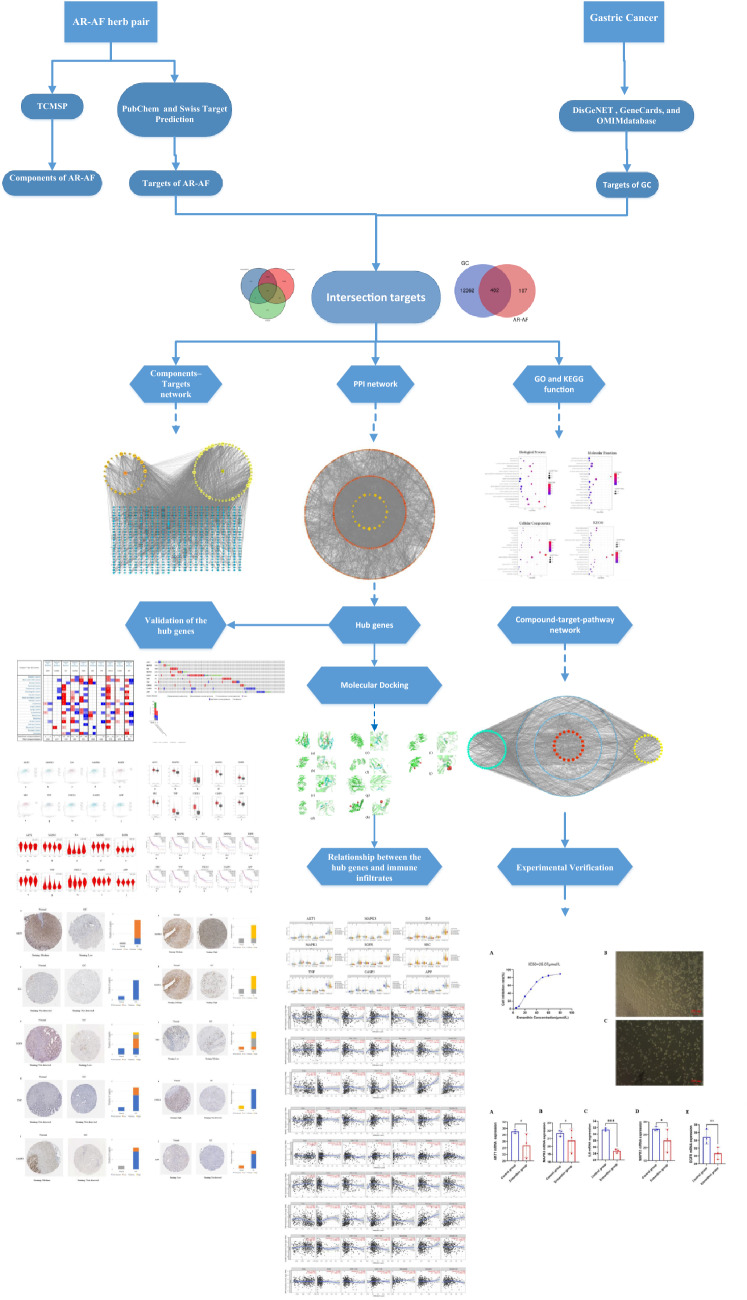
The protocol of our study procedures.

The development of network pharmacology provides new methods for elucidating multiple mechanisms of drug action^18^. The AR-AF herb pair has potential therapeutic effects on GC; however, the associations between AR-AF and GC have not been thoroughly studied and require further research. The present study was designed to delve into the mechanisms of AR-AF on GC using network pharmacology methods as an attempt to be referenced for subsequent pharmacological studies and clinical treatments of GC. The protocol of our study procedures is provided in Fig. 1.

## Methods

### Network pharmacology analysis

#### Main component screening

All constituents of the AR-AF herb pair were retrieved from the Traditional Chinese Medicine System Pharmacology Database and Analysis Platform (TCMSP)^19^ (http://lsp.nwu.edu.cn/tcmsp.php). As TCMSP suggests, the molecules with OB ≥ 30%^20^ and DL ≥ 0.05^21^ were preserved to display relatively better pharmacological properties and then screened out as candidate compounds for subsequent analysis. The PubChem database was used to obtain the structure data files (SDF) of the above candidate compounds and imported them into the SwissTargetPrediction database, taking the targets with a prediction score greater than 0 as the candidate targets. In addition, the “component-target” Network was constructed.

#### Mining GC-associated targets

Protein targets associated with GC were provided by DisGeNET (https://www.disgenet.org/home/)^22^, GeneCards (https://www.genecards.org/)^23^, and OMIM (https://omim.org/)^24^ with “gastric cancer” as the keyword. All the targets were limited to “homo sapiens”.

#### GO and KEGG enrichment analysis

The intersection of the two (the targets of GC and AR–AF) was taken to obtain the target set of AR-AF for the treatment of GC. Then the target set was imported into STRING (https://string-db.org/cgi/input.pl)^25^ to delve into protein–protein interactions, and “Organism” was set as “Homo sapiens”. A PPI interactive network was built and then visualized by Cytoscape software 3.7.2^26^. The NetworkAnalyzer tool was used for topology analysis. The three parameters of degree, betweenness centrality (BC), and closeness centrality (CC) were taken as reference standards, the top ten targets were selected as hub targets. The DAVID database (https://david.ncifcrf.gov/)^27^ was adopted to conduct GO and KEGG enrichment analyses.

### Validation of hub targets in different databases

Oncomine (https://www.Oncomine.org)^28^ was used to compare the differential expression of hub targets in GC tissues and normal gastric tissues. The genetic information of the correlation between mRNA expression and DNA methylation was conducted by cBioPortal tool (http://www.cbioportal.org/)^29^. The hub targets were input into the online tool GEPIA (http://gepia.cancer-pku.cn/index.html)^30^ to verify their mRNA expression level, pathological stages and overall survival (OS) in TCGA-STAD. Immunohistochemistry (IHC) was performed in the HPA (https://www.proteinatlas.org/)^31^.

### Molecular docking

The PDB database^32^ (https://www.rcsb.org/) was used to search the PDB files of the hub target proteins. The resolution of the crystal structures to achieve and carry out molecular modeling is 2.5–3.0 A. The PDB IDs corresponding to the hub target proteins selected in this study are AKT1 (PDB ID: 1UNQ^33^), MAPK3 (PDB ID: 6GES^34^), IL6 (PDB ID: 4O9H *To be published*), MAPK1 (PDB ID: 6OPH^35^), EGFR (PDB ID: 7JXP *To be published*), SRC (PDB ID: 6E6E^36^), TNF (PDB ID: 7KPA^37^), CXCL8 (PDB ID: 6WZM^38^), CASP3 (PDB ID: 6X8K *To be published*), and APP (PDB ID: 6ITU^39^). The SDF files of the main compounds with high degree were downloaded from the PubChem database (https://www.ncbi.nlm.nih.gov/pccompound/) as candidate docking medicinal ingredients, and the docking simulation calculation of molecules and target proteins was carried out by the molecular docking software AutoDock Vina 1.5.6^40^. The protein was processed using AutoDock Vina 1.5.6 software by separating the protein, adding nonpolar hydrogen, calculating the Gasteiger charge, assigning the AD4 type, and setting all the flexible bonds of the small molecule ligands to be rotatable. The receptor proteins were set as rigid docking, and a genetic algorithm was selected with the maximum eval number as the medium. Before docking, ChemBioDraw 3D was used to generate 3D chemical structures and minimize energy for all selected ligands. Grid box parameters can be seen in Supplementary Table S3. The docking results were obtained by running autogrid4 and autodock4, revealing the binding energy. The relatively stable results of molecular docking were selected to draw a 3D map with PyMol software^41^.

The best docking poses were identified as those showing the smallest root mean square deviation (RMSD) between the predicted conformation and the observed X-ray crystallographic conformation. Models with an RMSD ≤ 4 Å were considered reliable and those with an RMSD ≤ 2 Å were considered accurate^42^. In this paper, models with an RMSD ≤ 2 Å were considered.

### Immune cell infiltrates of hub targets

The efficacy of immune checkpoint inhibitors is affected by many factors. To illuminate the underlying mechanism of the immune microenvironment of GC, we used the TIMER online tool (https://cistrome.shinyapps.io/timer/)^43^ to investigate the effect of somatic cell copy number (CNA) of the hub targets on different kinds of immune cell infiltration and the association between hub targets and the immune infiltrate level.

### Experimental verification in vitro

#### Cell cultures and cell viability measurements

AGS cells were cultured in F12K medium (Gibco, MD, USA) supplemented with 5% fetal bovine serum (FBS) (Gibco, MD, USA) and 100 U of penicillin G with 100 μg of streptomycin per ml. Cells were incubated at 37 °C under 5% CO2 in a humidified atmosphere. AGS cells were inoculated into 96-well plates at a cell density of 5 × 10^4^ cells/well and cultured for 24 h. Different concentrations of eremanthin (0, 5, 10, 20, 30, 40, 50, 60, 80 μmol/L) were added for 24 h. The inhibition rate of the cells was determined using the CCK8 kit to explore whether eremanthin had a toxic effect on the cells. Finally, optical density (OD) measurements were performed at 560 nm with microplate reader.

#### Quantitative real-time polymerase chain reaction (qRT–PCR)

According to the molecular docking results, eremanthin with a good docking effect was selected as the main compound for experimental verification. On the basis of network pharmacology, we selected the top five targets, AKT1, MAPK3, IL6, MAPK1, and EGFR to explore the mechanism. Total RNA was extracted from cultured cells using TRIzol according to the manufacturer’s instructions and then reverse transcribed using the RevertAid RT Reverse Transcription Kit (Invitrogen, K1691). The primer sequences used for qRT–PCR are listed in Table 1. qRT–PCR was performed using a StepOne Real-Time PCR system (Applied Biosystems).

**Table 1:** Real-time polymerase chain reaction primers.

Gene	Sequence (5′-3′)
AKT1-F	GGTTAGCCACTCTATCGCCATGAC
AKT1-R	CCACAAGCCATTCTCCACTCCAC
MAPK3-F	TTCTGTTGCTGTCTCCTCCTCTCC
MAPK3-R	AGGTGATTAGGGTGTGGCTCTGAG
IL6-F	GACAGCCACTCACCTCTTCAGAAC
IL6-R	AAGCCTACCCACCTCCTTTCTCAG
MAPK1-F	TCCTGCTGCCTTCACTCACTCC
MAPK1-R	GCCTGCTGCTCCACAGAGAATG
EGFR-F	AACAATGGCTGAGCGTGGTAGATG
EGFR-R	GGAGTGAACAAGAACGGGCAGAC
β-ACTIN-F	CAGATGTGGATCAGCAAGCAGGA
β-ACTIN-R	CGCAACTAAGTCATAGTCCGCCTA

#### Statistical analysis

The experimental results were statistically analyzed by GraphPad Prism software. A single-factor ANOVA was used to compare the differences in experimental data among groups, and P < 0.05 revealed significant difference.

## Results

### Network pharmacology analysis

#### Main component screening

106 compounds were screened out in total following the OB ≥ 30% and DL ≥ 0.05 criteria, including 46 AR and 60 AF. Supplementary Table S1 lists the 106 main compounds. With the highest degrees, eremanthin, cynaropicrin, and aceteugenol were considered as the vital main compounds.

#### Component-target network construction

**Figure 2:**
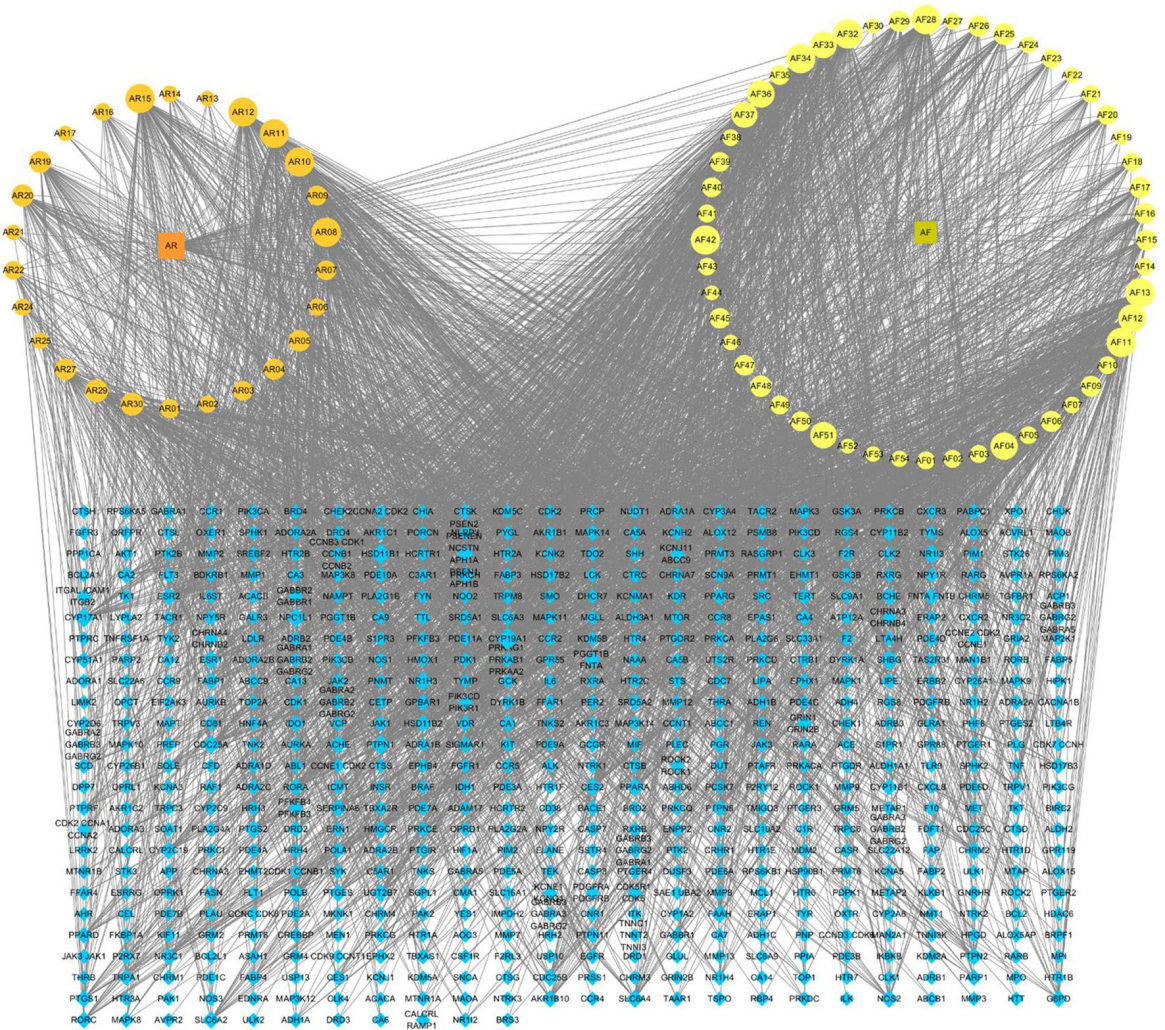
Compound-target network. Orange circle nodes represent the main components of AR, and yellow circle nodes represent the main components of AF. Blue rhombus nodes represent the targets of AR-AF.

**Figure 3:**
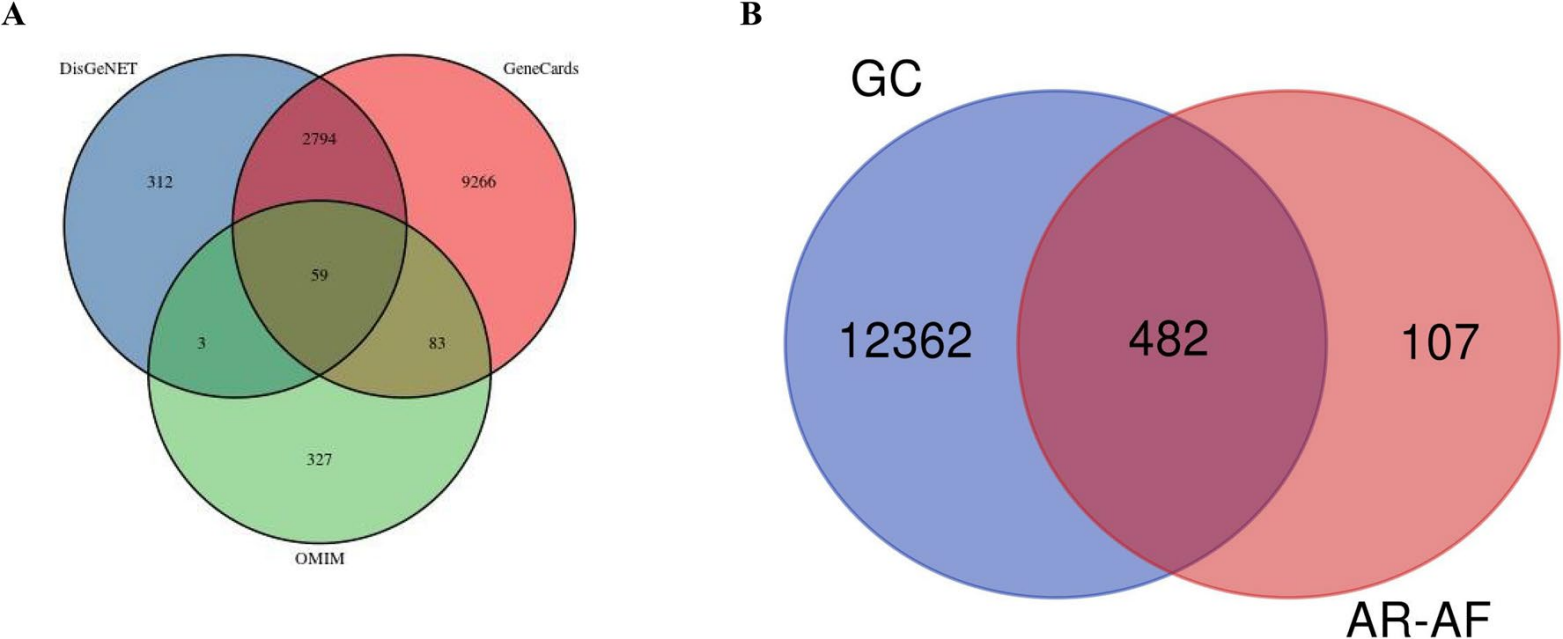
Venn diagram of target genes. (**A**) GC-related targets in different databases. (**B**) Venn diagram of AR-AF and GC targets.

A total of 1135 potential targets of AR and 2148 potential targets of AF were obtained from the database prediction and screening. We established a compound-target network with 667 nodes and 3361 edges (Fig. 2). In this network, the relationships between the main compounds and compound targets as well as the potential pharmacological effects of AR-AF were visually illustrated. We identified 12,202, 3168 and 472 GC-related targets from GeneCards, DisGeNET and OMIM, respectively, which resulted in 12,844 targets after merging and removing duplicate targets (Fig. 3A). A total of 482 target genes were identified to be affected by GC and regulated by AR-AF, which are more critical to treat GC (Fig. 3B).

#### GO and KEGG enrichment analysis

**Figure 4:**
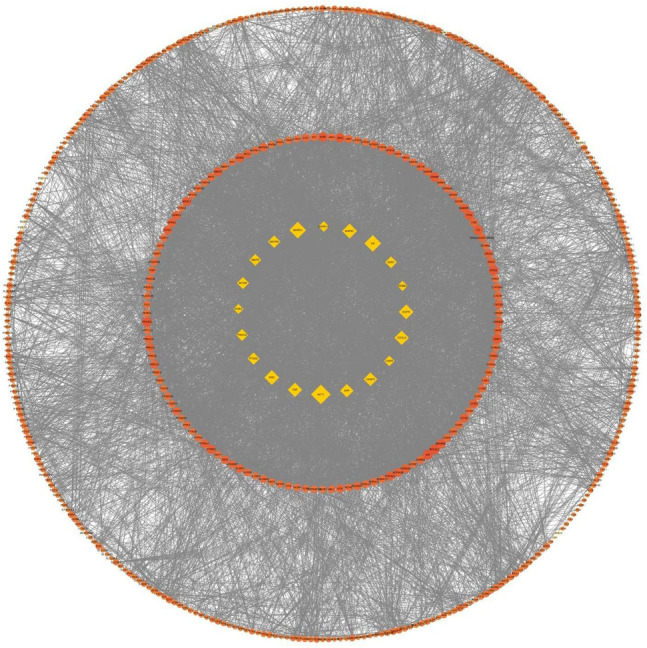
The PPI network. Yellow diamonds represent the top 20 genes in all nodes with degree ≥ 83. Orange circles represent the genes with degree < 83. Node size and color were set to reflect the degree value. The greater the degree value is, the darker the color and the larger the node.

To identify the most highly connected nodes from others, PPI network analysis was conducted. This network is illustrated in Fig. 4, covering 482 nodes and 7885 edges. AR-AF exerted its therapeutic effects on GC through multiple protein targets. The nodes with the highest degrees included AKT1, MAPK3, IL6, MAPK1, EGFR, SRC, TNF, CXCL8, CASP3, and APP.

**Figure 5:**
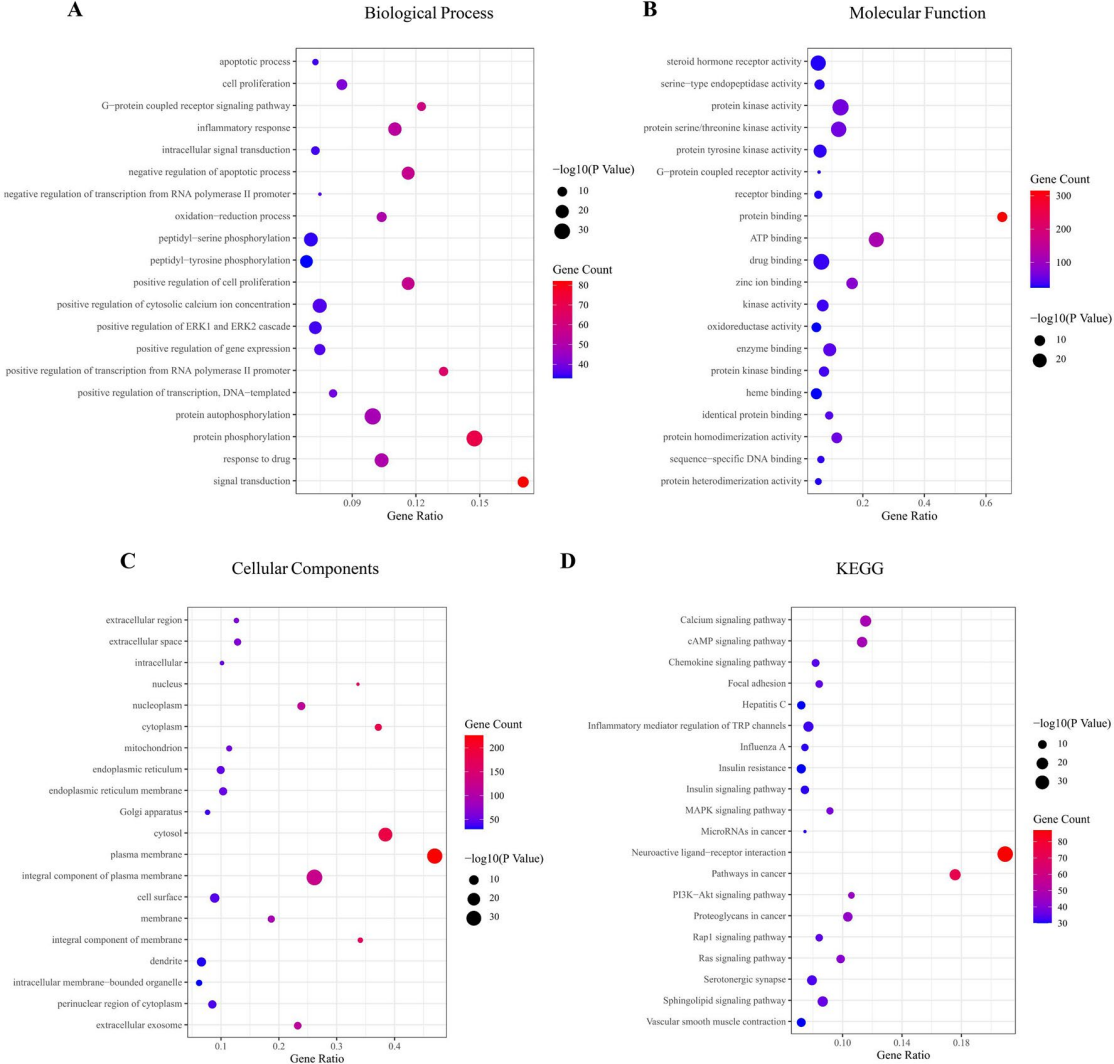
Bubble diagram for GO and KEGG enrichment analysis. (**A**) Biological processes for the major targets. (**B**) Molecular function for the major targets. (**C**) Cellular components for the major targets. (**D**) Represents KEGG for the major targets. The bubble size represents the number of enriched genes, and the bubble color difference represents the significant magnitude of target gene enrichment.

**Figure 6:**
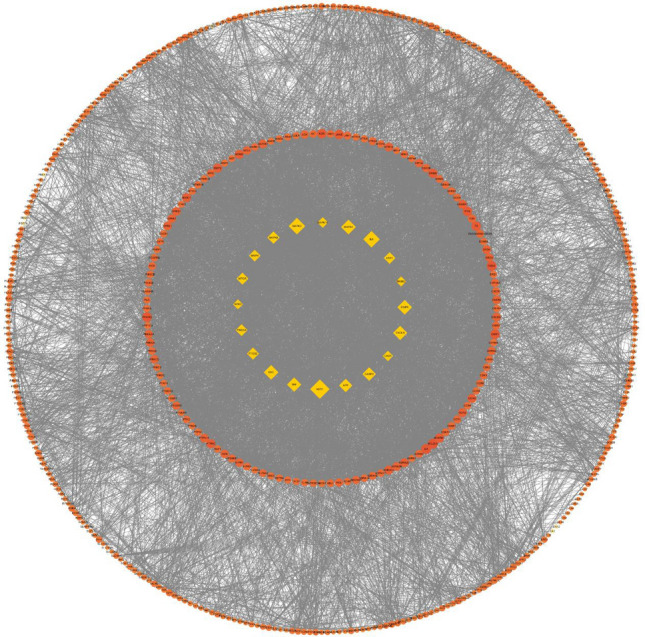
Compound-target-pathway network. Red hexagonal nodes represent pathways, blue rectangle nodes represent targets, yellow circle nodes represent AR, and green circle nodes represent AF.

To clarify the function of the estimated protein targets, GO and KEGG enrichment analyses were conducted. The top 20 noticeably enriched GO and KEGG terms are listed in Fig. 5. As suggested from the results, the targets of AR-AF displayed tight relations to the major biological process, which included signal transduction, protein phosphorylation, positive regulation of transcription from the RNA polymerase II promoter, and the G-protein coupled receptor signaling pathway. Through KEGG enrichment analysis, AR-AF probably exerted therapeutic effects on GC by regulating signaling pathways, which included the cAMP signaling pathway, and PI3K-Akt signaling pathway. To illustrate the relationship between the AR-AF herb pair and their corresponding compound targets and GC targets, a compound-target-pathway network was built (Fig. 6).

### Molecular docking

**Figure 7:**
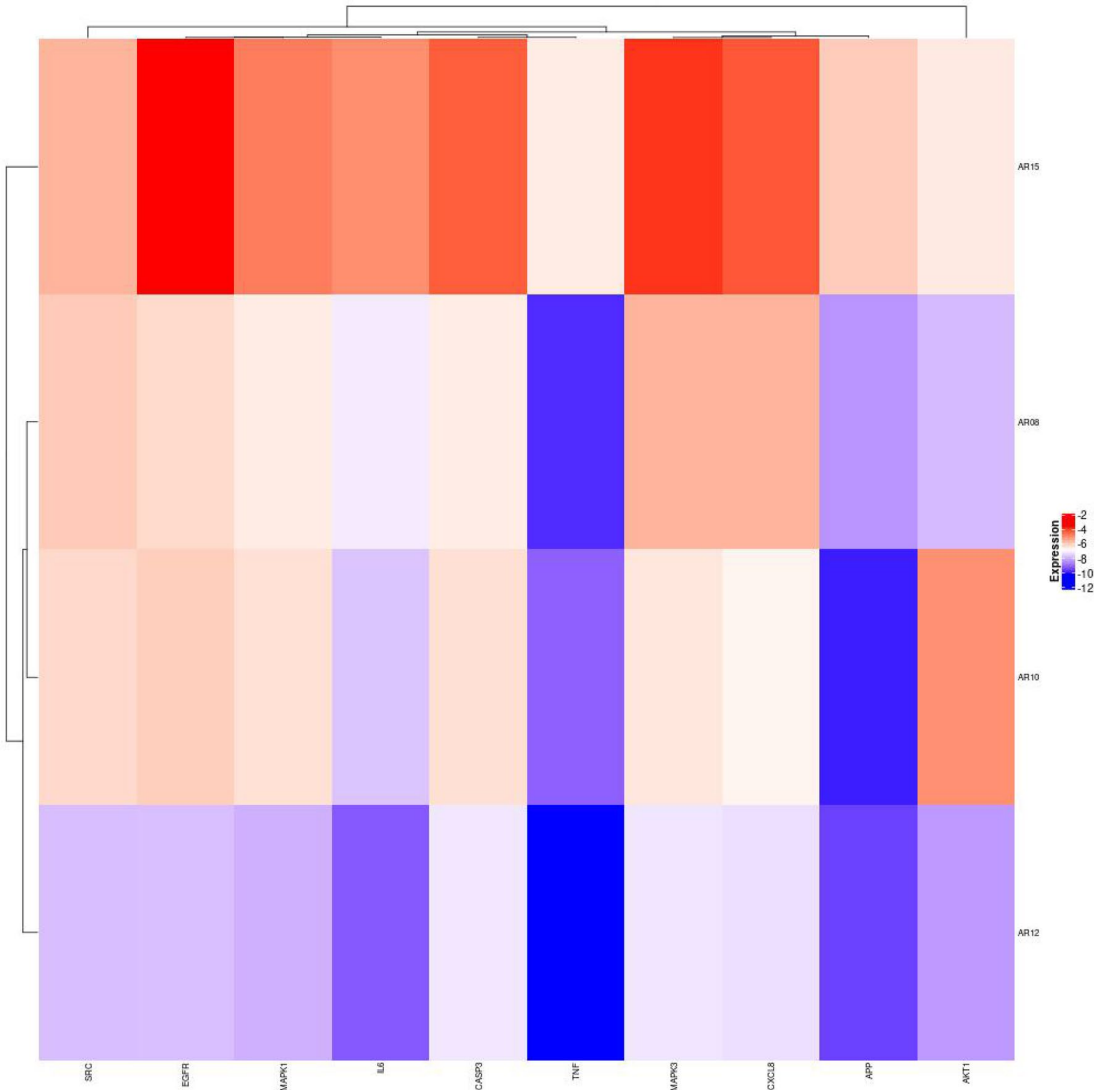
Heatmap of binding affinity. The bluer the color is, the more stable the binding force. AR10 stands for cynaropicrin, AR08 stands for 2- [(2r, 4ar, 8as)-4a-methyl-8-methyl-decalin-2-yl] acrylic acid, AR12 stands for eremanthin, and AR15 stands for aceteugenol.

**Figure 8:**
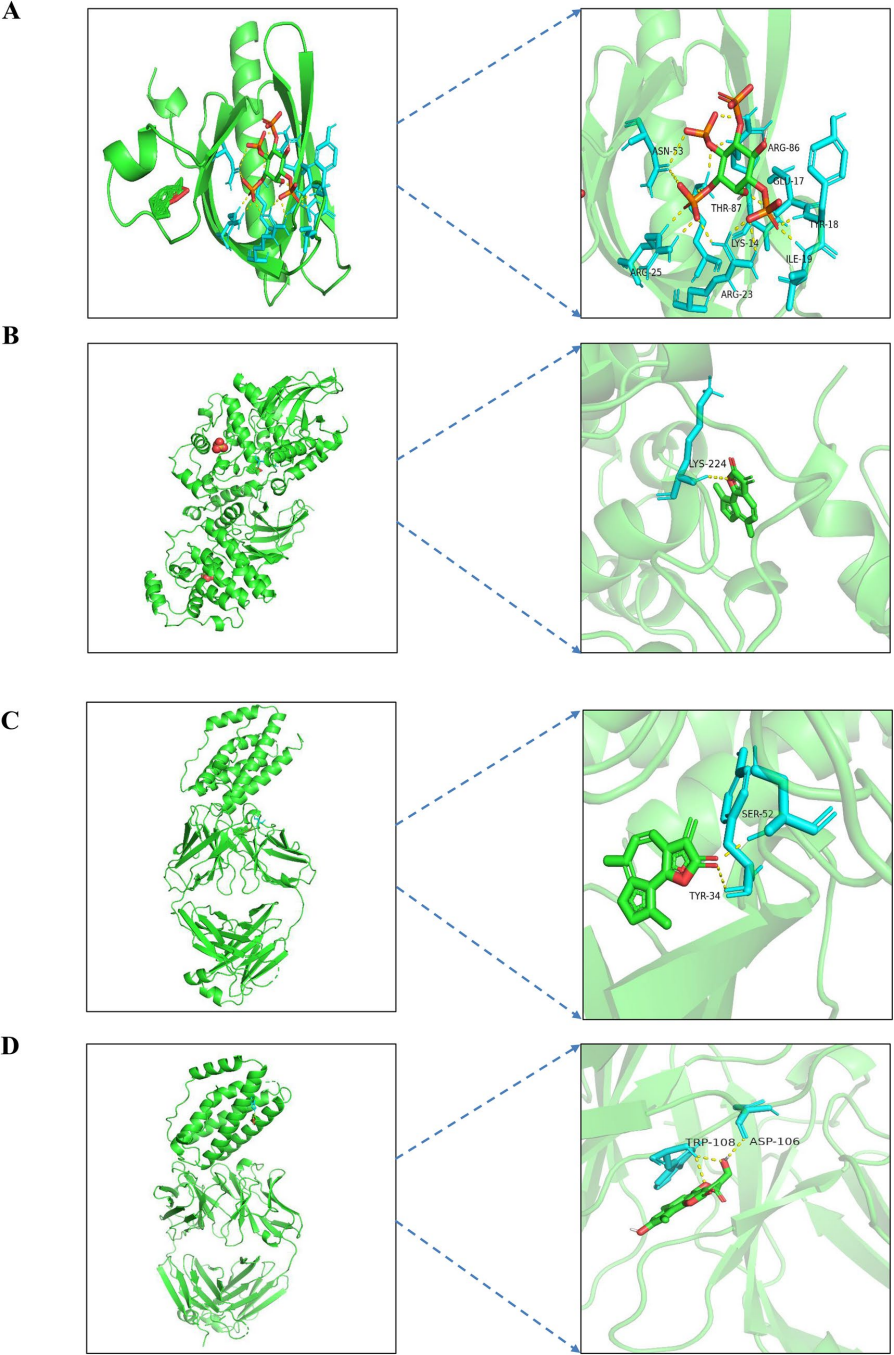

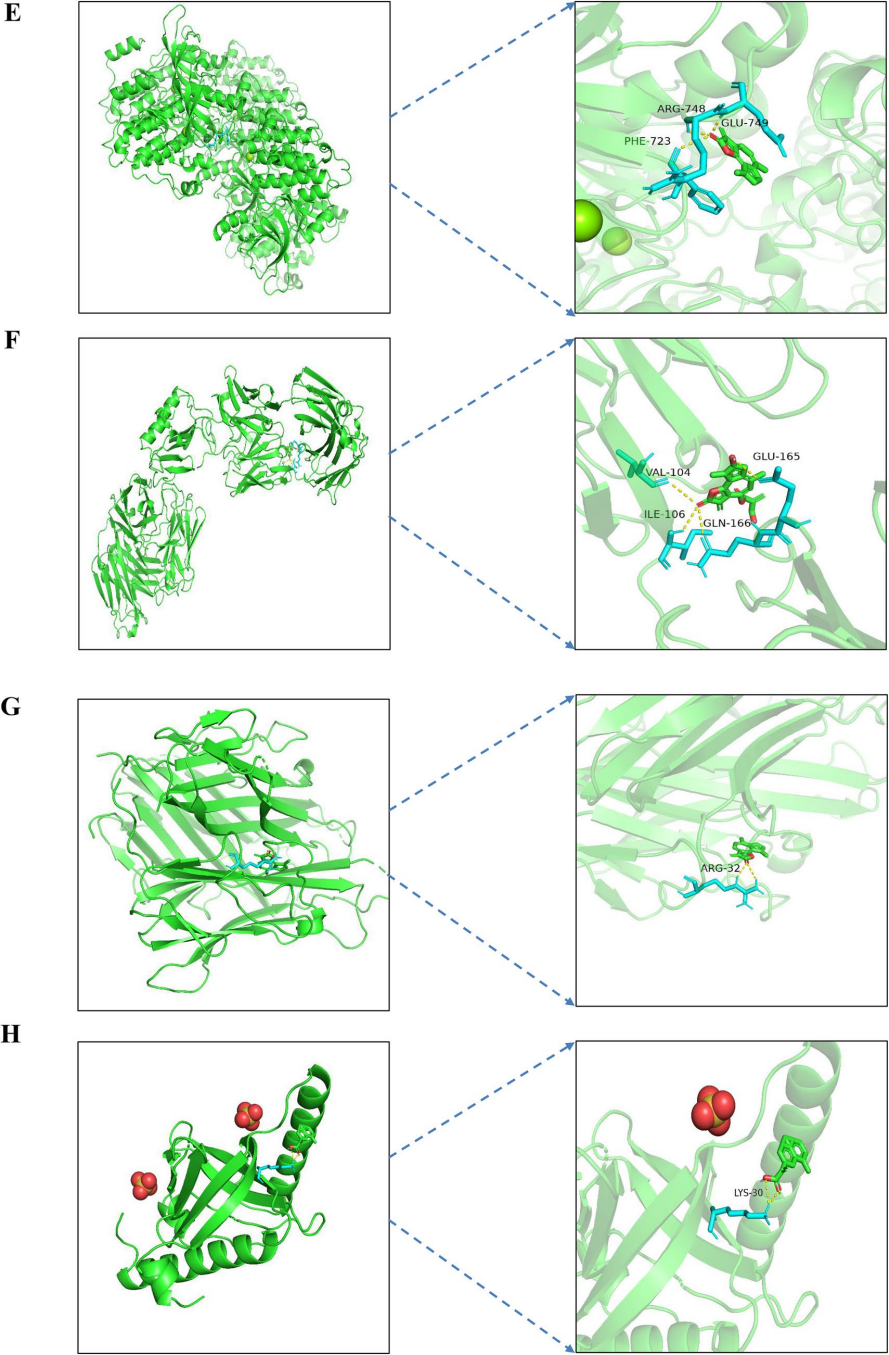

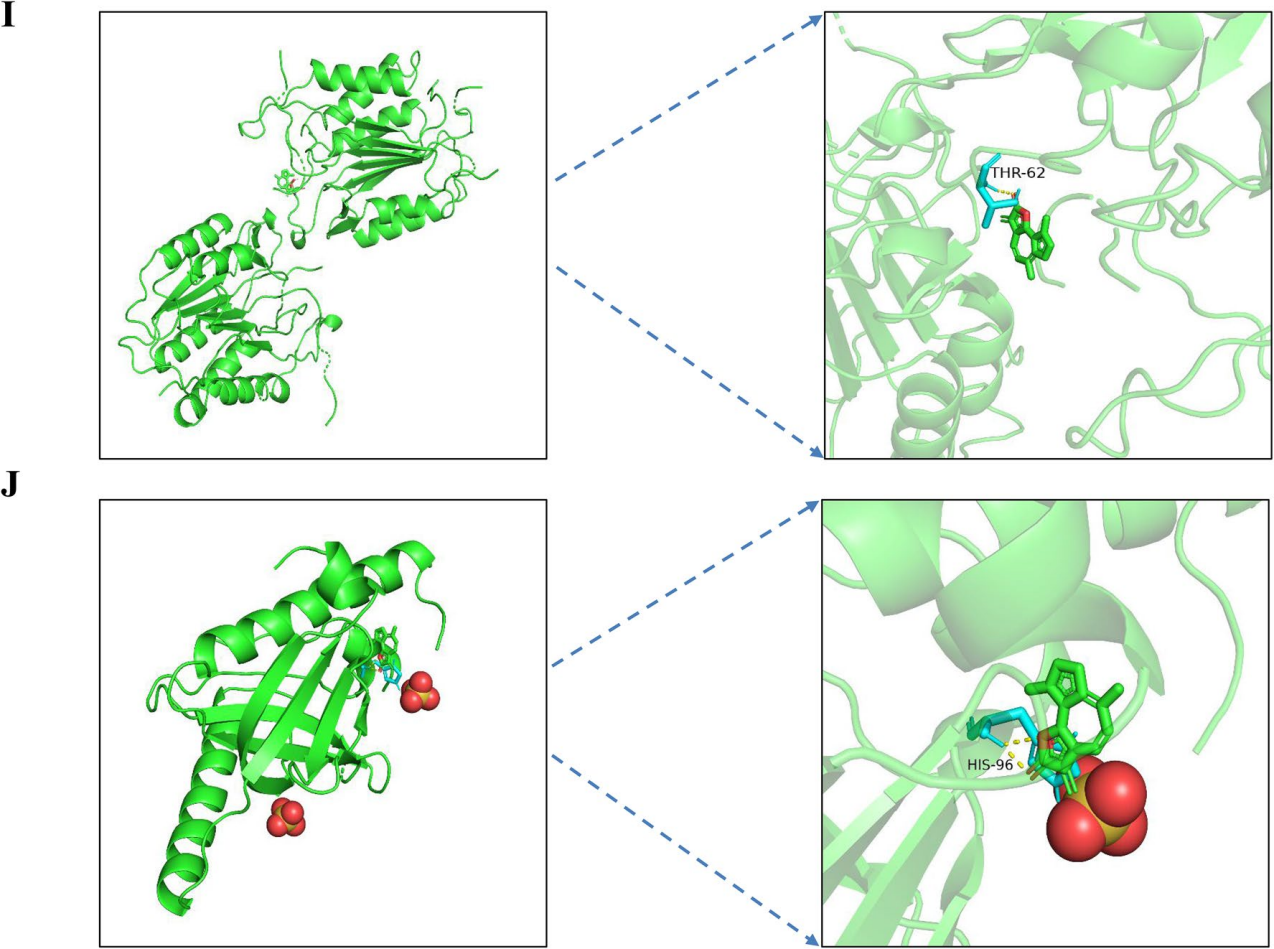
Schematic diagram of docking between eremanthin and proteins. Molecular models of eremanthin binding to the predicted target proteins (**A**) 1UNQ, (**B**) 6GES, (**C**) 4O9H, (**D**) 6OPH, (**E**) 7JXP, (**F**) 6E6E, (**G**) 7KPA, (**H**) 6WZM, (**I**) 6X8K, and (**J**) 6ITU.

Molecular docking is mainly used to investigate the molecular recognition between small molecules and target proteins and to identify the binding of the molecules with the active sites of the target^44^. If the binding energy of docking is negative, it indicates that the small molecule can effectively and autonomously bind to the target protein. It is generally believed that the lower the energy when the conformation of ligand-receptor binding is stable, the more likely the effect will be^45^. A binding energy <  − 5 kcal mol^−1^ indicates good binding activity, and a binding energy <  − 7 kcal mol^−1^ indicates strong binding activity. The results of molecular docking were plotted as a heatmap, as shown in Fig. 7. It can be seen from the figure that cynaropicrin, 2-[(2r, 4ar, 8as)-4a-methyl-8-methyl-decalin-2-yl] acrylic acid, and eremanthin with IL6, TNF, and APP all had good activities, and the binding affinity is shown in Supplementary Table S2. The docking results of eremanthin with good activity were selected and visualized by PyMol software. In general, the calculated interactions of experimental structures of ligand–protein complexes have two different regions of high probability density. The first identifiable geometry is described by the relatively close distance between the centers of the aromatic rings (3.5–4.5 Å) between the ring plane normal vector, with a range of parameters corresponding to parallel aromatic hydrocarbon accumulation. The second dense region described by the longer distance and the near right angle of 5–6 Å corresponds to the vertical aromatic interaction^46^. In this paper, the interaction between ligands and proteins is aromatic accumulation. Eremanthin formed π–π interactions with ASN-53 (3.0 Å), ARG-25 (2.1 Å), LYS-14 (2.0 Å), TYR-18 (1.8 Å), and AGR-86 (1.9 Å) to form hydrogen bonds and with ARG-23 (3.0 Å), Glu-17 (2.7 Å), ILE-19 (1.8 Å), and THR-87 (2.5 Å) to form π–π interactions that resulted in binding to AKT1 (1UNQ) (Fig. 8A). Similarly, eremanthin was predicted to pair into the binding pocket of MAPK3 (6GES) by hydrogen bonding to LYS-224 (4.0 Å) (Fig. 8B). Eremanthin was also predicted to dock in the binding pocket of IL6 (4O9H) by 2 hydrogen bonds to TYR-34 (2.3 Å) and SER-53 (2.1 Å) (Fig. 8C). Eremanthin formed a π–π interaction with ASP-106 (3.3 Å) through hydrogen bonding with TRP-108 (2.8 Å) in the binding pocket of MAPK1 (6OPH) (Fig. 8D). Eremanthin was in the binding pocket of EGFR (7JXP) by multiple hydrogen bonds with PHE-723 (3.4 Å), ARG-748 (3.0 Å), and GLU-749 (2.7 Å) (Fig. 8E). Eremanthin bonded to SRC (6E6E) through hydrogen bonds with VAL-104 (4.1 Å), ILE-106 (4.4 Å), and GLN-166 (4.3 Å) and through a π–π interaction with GLU-165 (2.0 Å) (Fig. 8F). Finally, eremanthin was attached to the binding pocket of TNF (7KPA), CXCL8 (6WZM), CASP3 (6X8K), and APP (6ITU) by hydrogen bonding with ARG-32 (2.4 Å), LYS-30 (2.1 Å), THR-63 (2.9 Å), and HIS-96 (1.6 Å), respectively (Fig. 8G–J). Overall, these results provide further evidence that the hub proteins act as eremanthin targets in GC.

### Validation of hub targets in different databases

**Figure 9:**
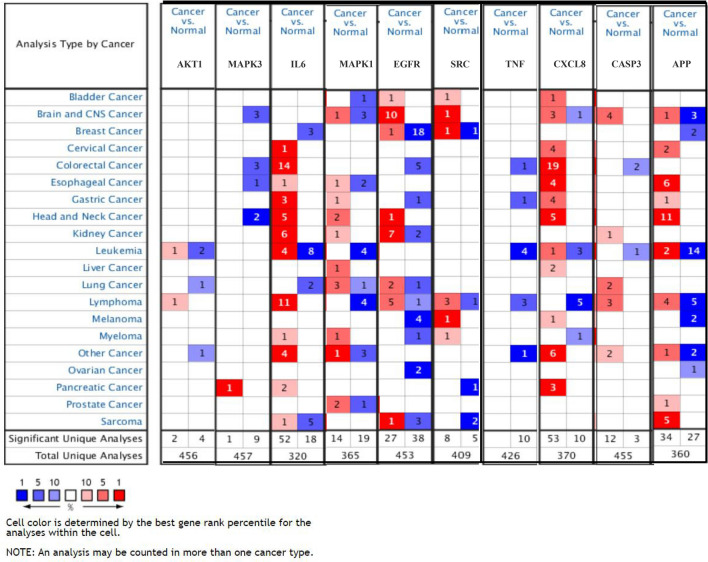
mRNA expression levels of hub targets. The red box indicates the overexpression of the gene in tumor tissues, while the blue box indicates the downregulation of the targets. The intensity of expression is expressed in shades of color.

Oncomine showed that IL6, MAPK1, CXCL8, and APP were all expressed statistically significantly in GC. Although AKT1, MAPK3, SRC, and CASP3 were not expressed in GC, the analysis showed that they were expressed in other tumors (Fig. 9).

**Figure 10:**
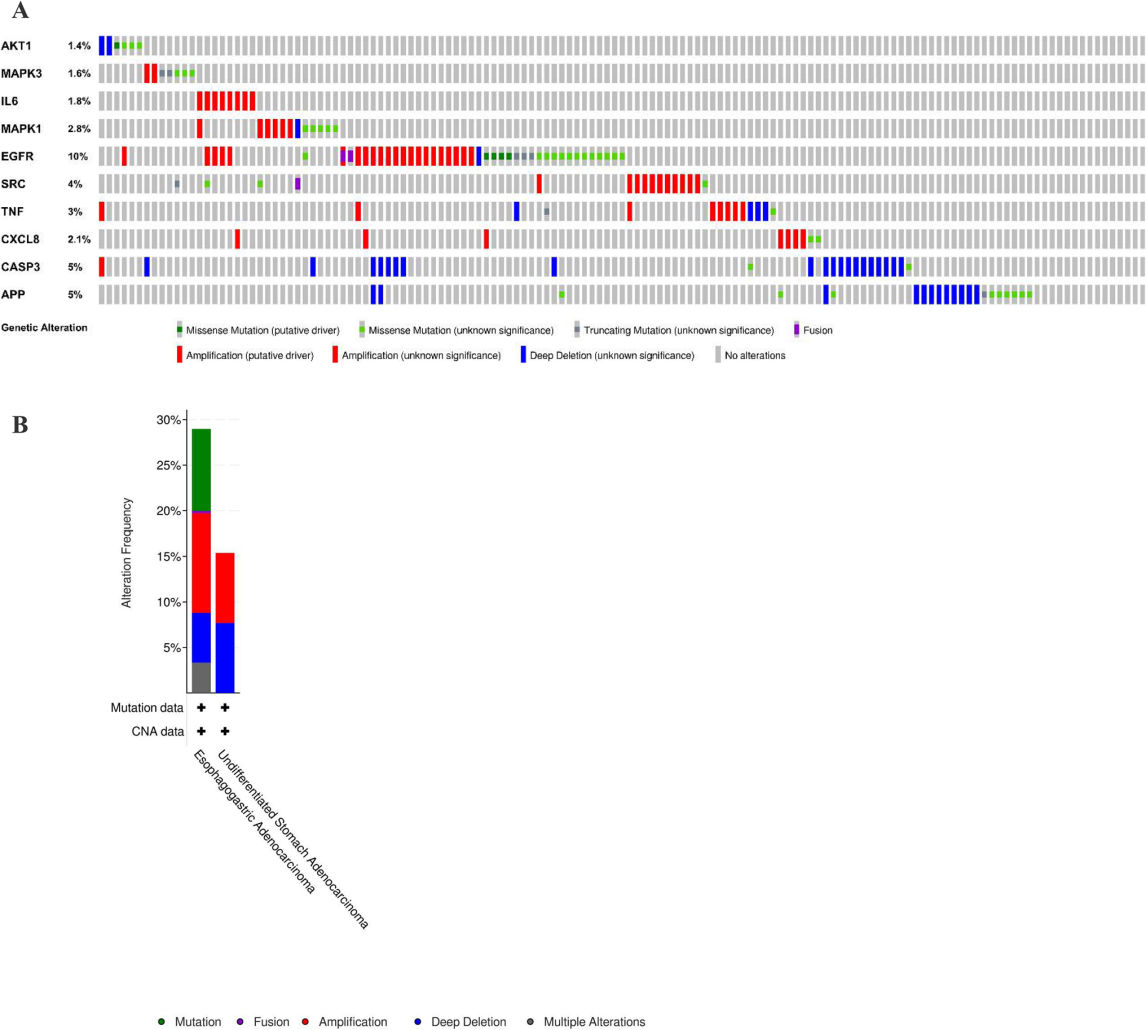

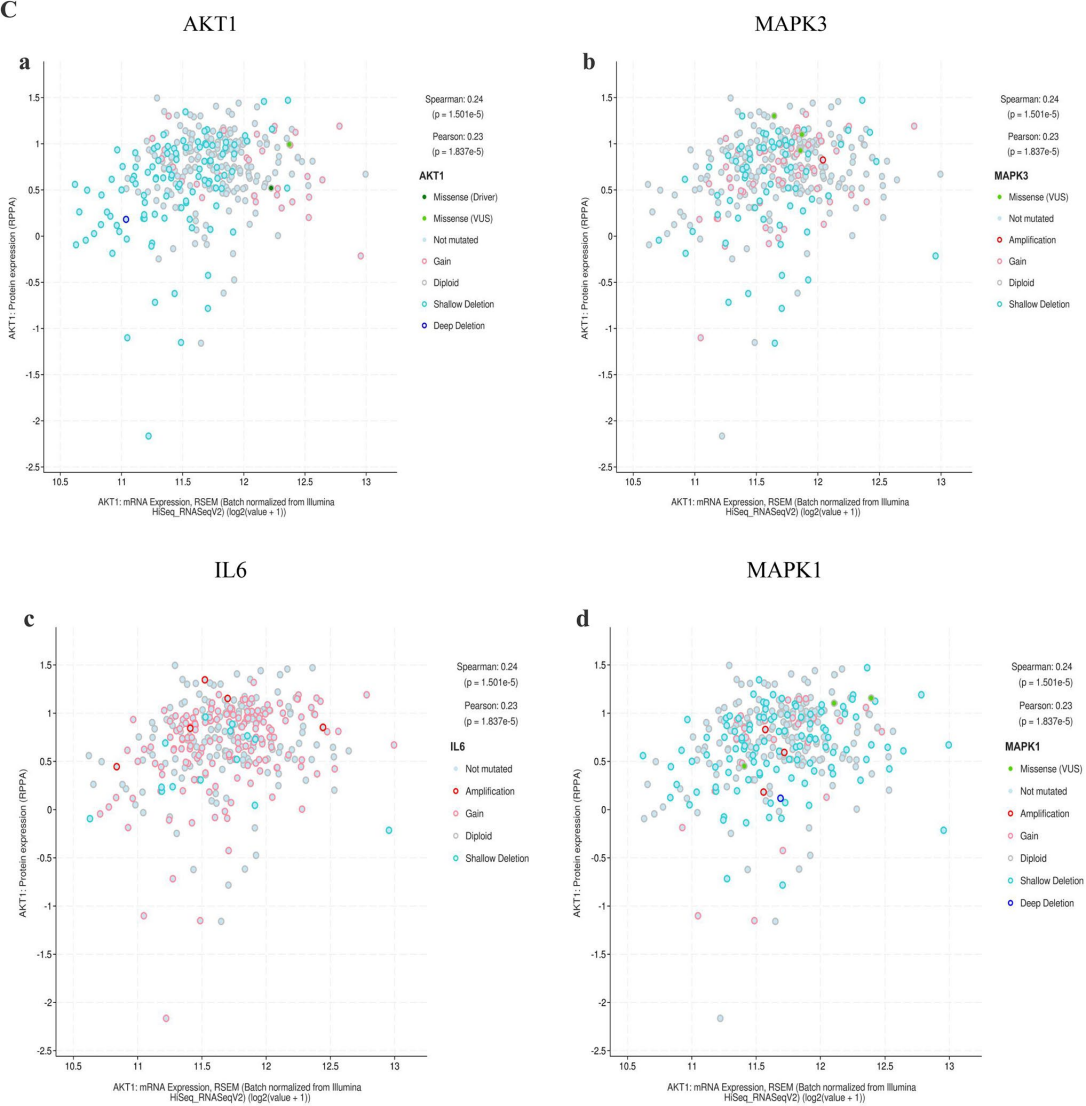

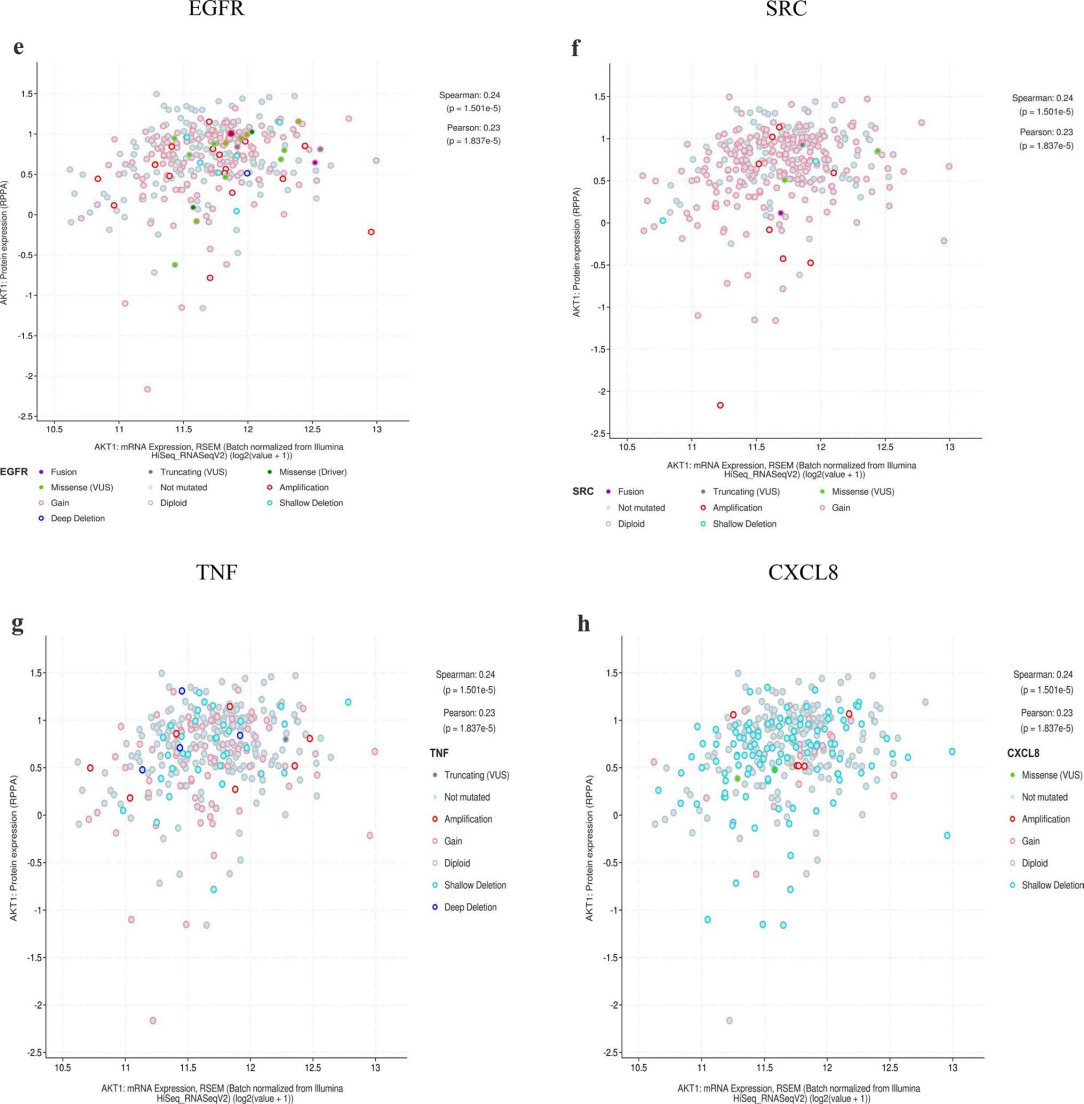

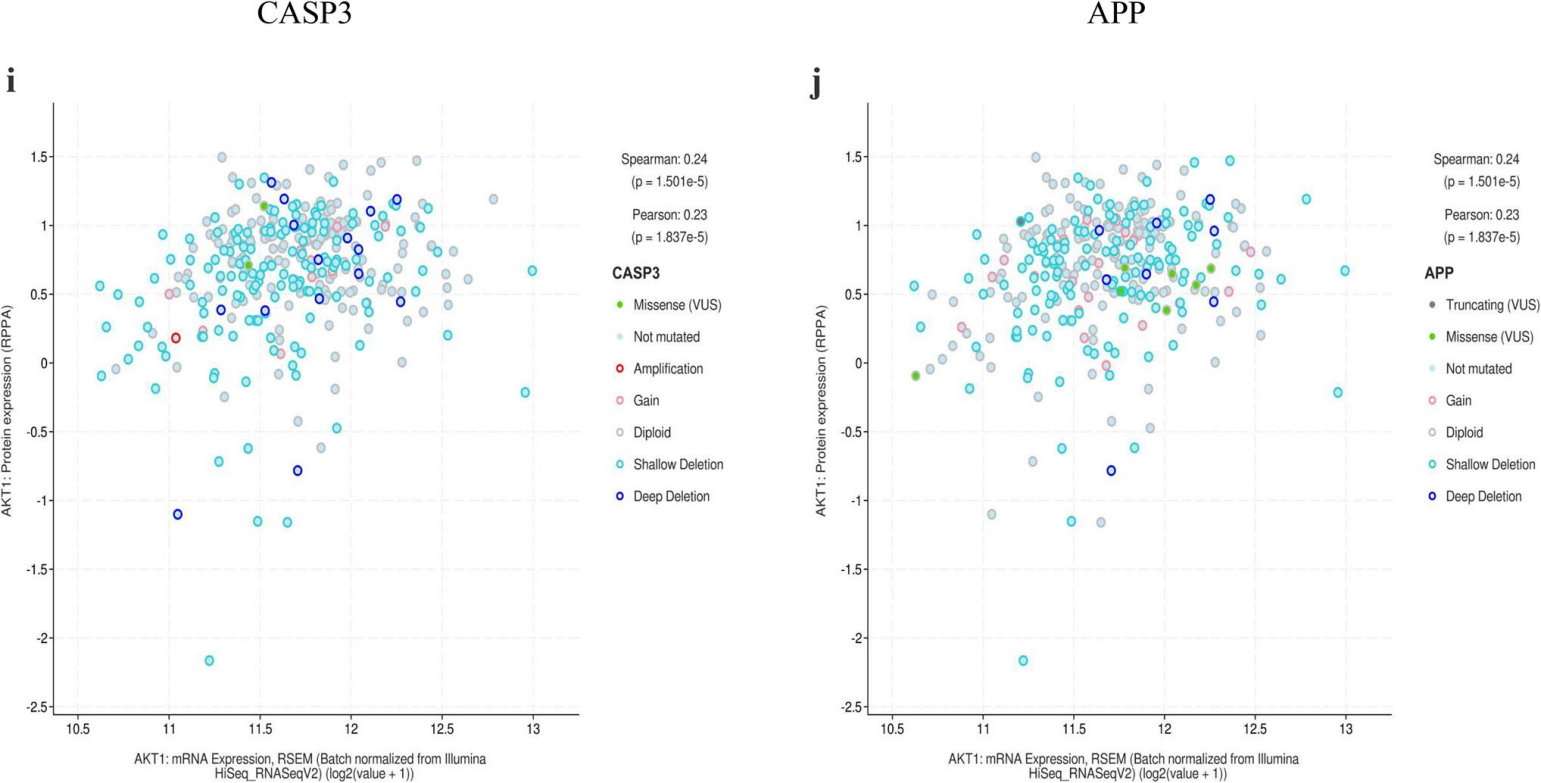
Genetic information of hub targets. (**A**) Data from TCGA of gastric adenocarcinoma showed that 124 of 434 patients (29%) had genetic mutations in these targets. (**B**) The diagram shows an overview of the genetic variation of the hub targets. (**C**) The diagram shows the correlation between the mRNA and protein levels of (a) AKT1, (b) MAPK3, (c) IL6, (d) MAPK1, (e) EGFR, (f) SRC, (g) TNF, (h) CXCL8, (i) CASP3, and (j) APP.

The cBioPortal tool showed that 124 of 434 patients with gastric adenocarcinoma (29%) had genetic mutations in these targets (Fig. 10A). An overview of the genetic variation of hub targets was also analyzed (Fig. 10B). Figure 10C shows the correlation between the mRNAs and proteins of the hub targets.

**Figure 11:**
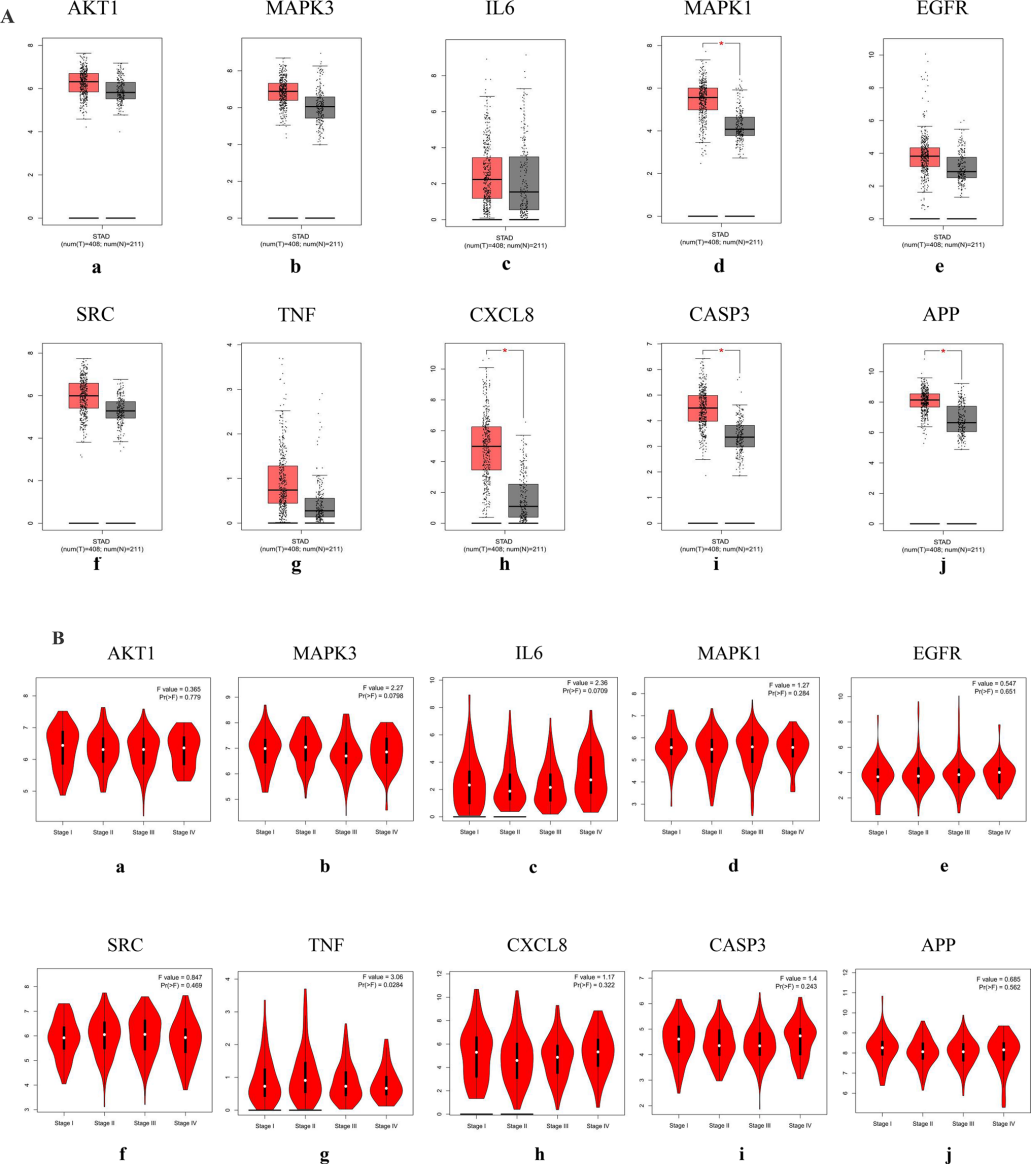

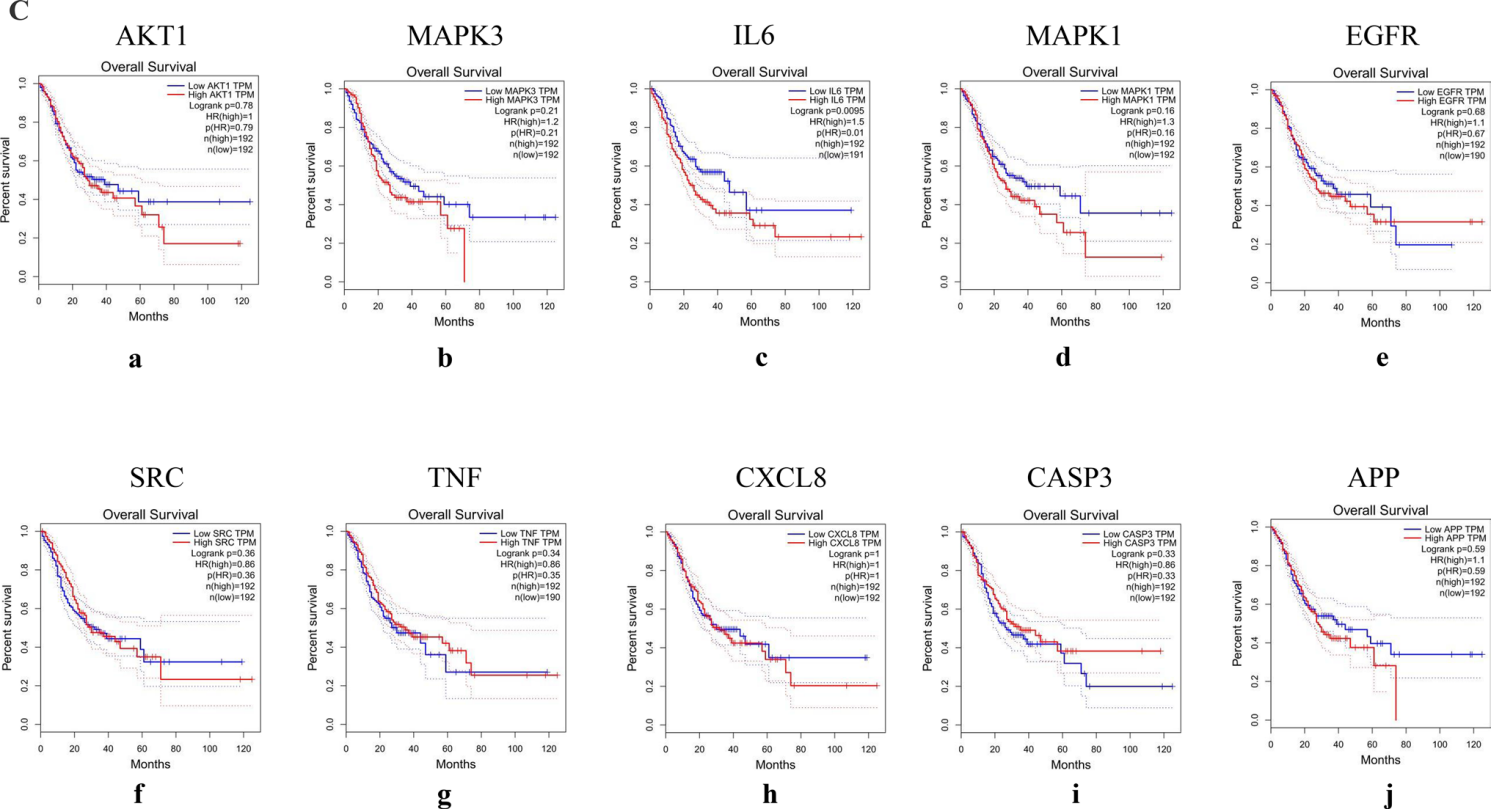
mRNA expression level, pathological stage, and OS of hub targets. (**A**) Box plots showing the mRNA expression levels of (a) AKT1, (b) MAPK3, (c) IL6, (d) MAPK1, (e) EGFR, (f) SRC, (g) TNF, (h) CXCL8, (i) CASP3, and (j) APP. Red represents tumor, gray represents normal. (**B**) The violin diagram shows the stage plot of mRNA expression level and pathological stage in the GEPIA database. (**C**) The line charts show the OS of hub targets. The survival curve comparing the patients with high (red) and low (blue) expression in GC.

The mRNA levels of MAPK1, CXCL8, and CASP3 were significantly higher in GC tissues (Fig. 11A). The levels of TNF changed significantly with pathological stage and increased significantly in stage II (Fig. 11B). The Kaplan–Meier survival curve (Fig. 11C) showed that the prognostic value of IL6 was significantly different (P < 0.05).

**Figure 12:**
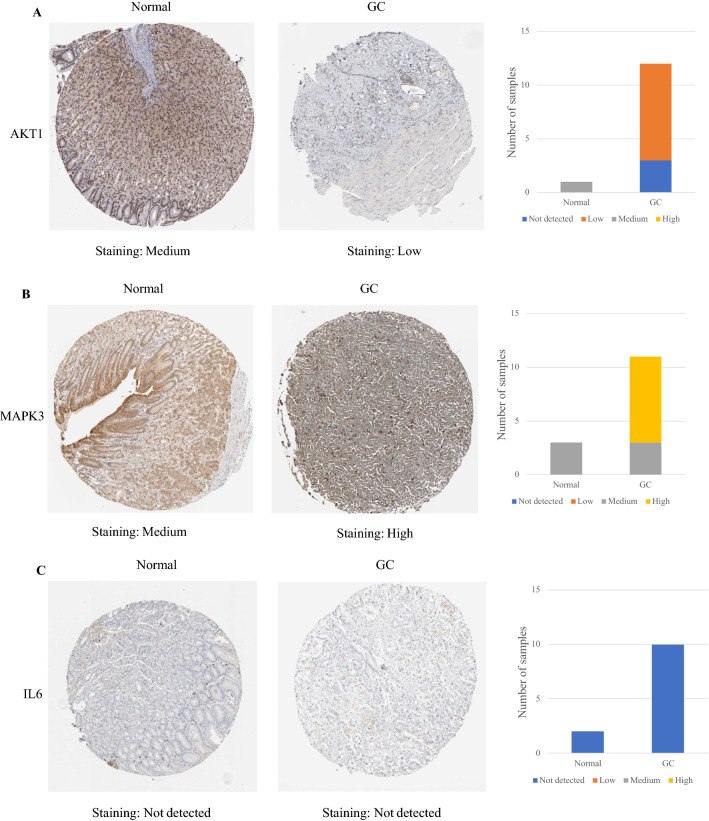

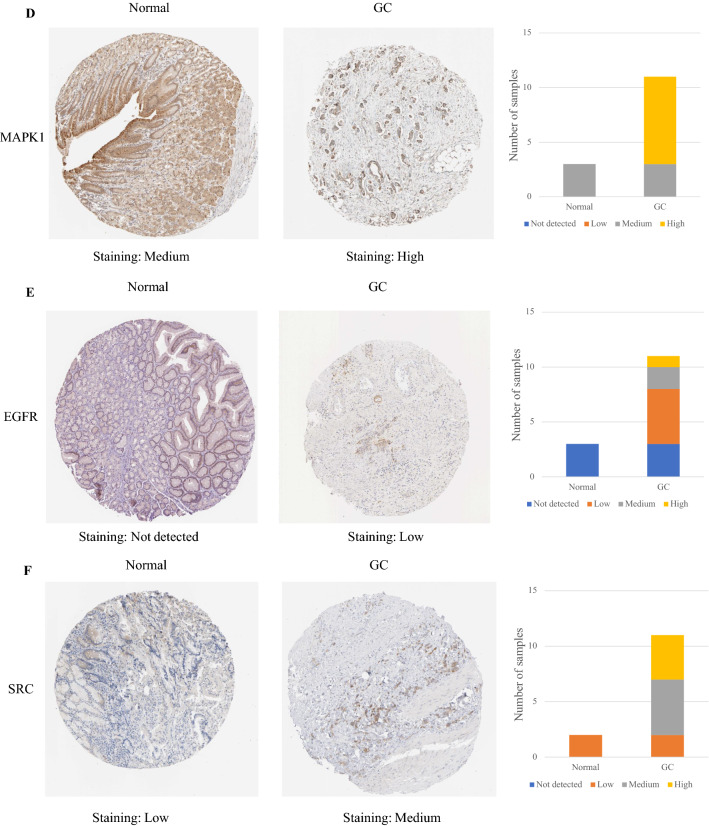

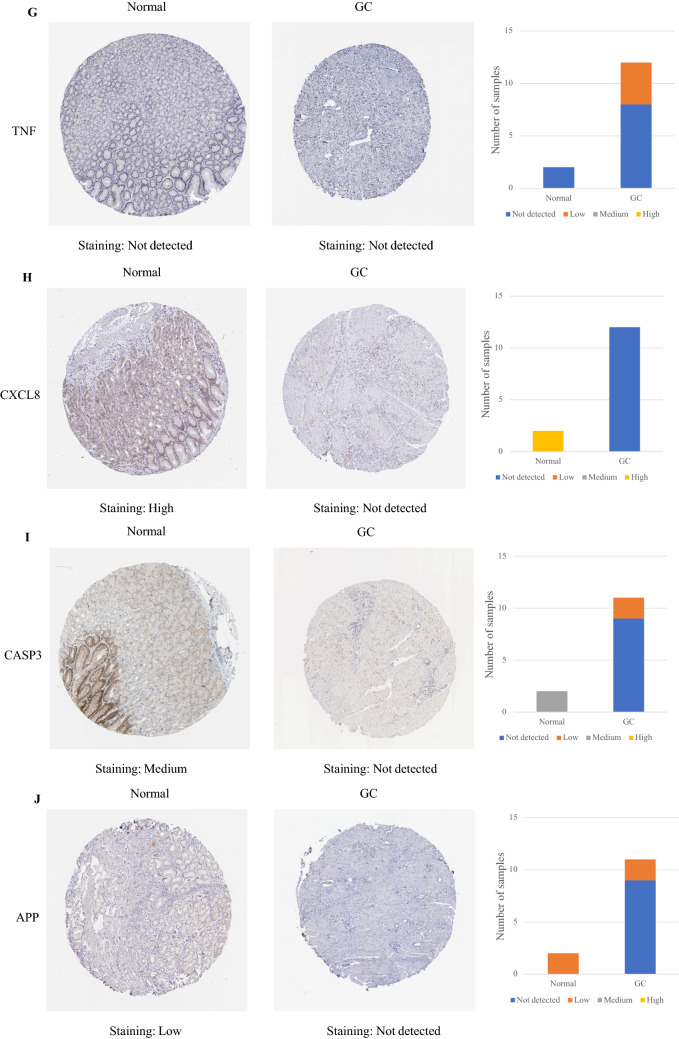
IHC of hub targets in the HPA. Representative immunohistochemistry images of (**A**) AKT1, (**B**) MAPK3, (**C**) IL6, (**D**) MAPK1, (**E**) EGFR, (**F**) SRC, (**G**) TNF, (**H**) CXCL8, (**I**) CASP3, and (**J**) APP in GC and noncancerous stomach tissues. The staining strengths were annotated as Not detected, Low, Medium, and High. The bar plots indicate the number of samples with different staining strengths.

The protein expression of IL-6 and TNF between gastric cancer tissue and normal gastric tissue was the same, indicating that there was no difference. In contrast, the other eight hub targets were expressed to different degrees between normal gastric tissue and gastric cancer tissue, which means that there were significant differences. Compared with normal gastric tissue, the expression levels of MAPK3, MAPK1, EGFR, and SRC were increased in GC tissue, while the expression levels of AKT1, CXCL8, CASP3, and APP were decreased in GC tissue (Fig. 12).

### Immune cell infiltrates of hub targets

**Figure 13:**
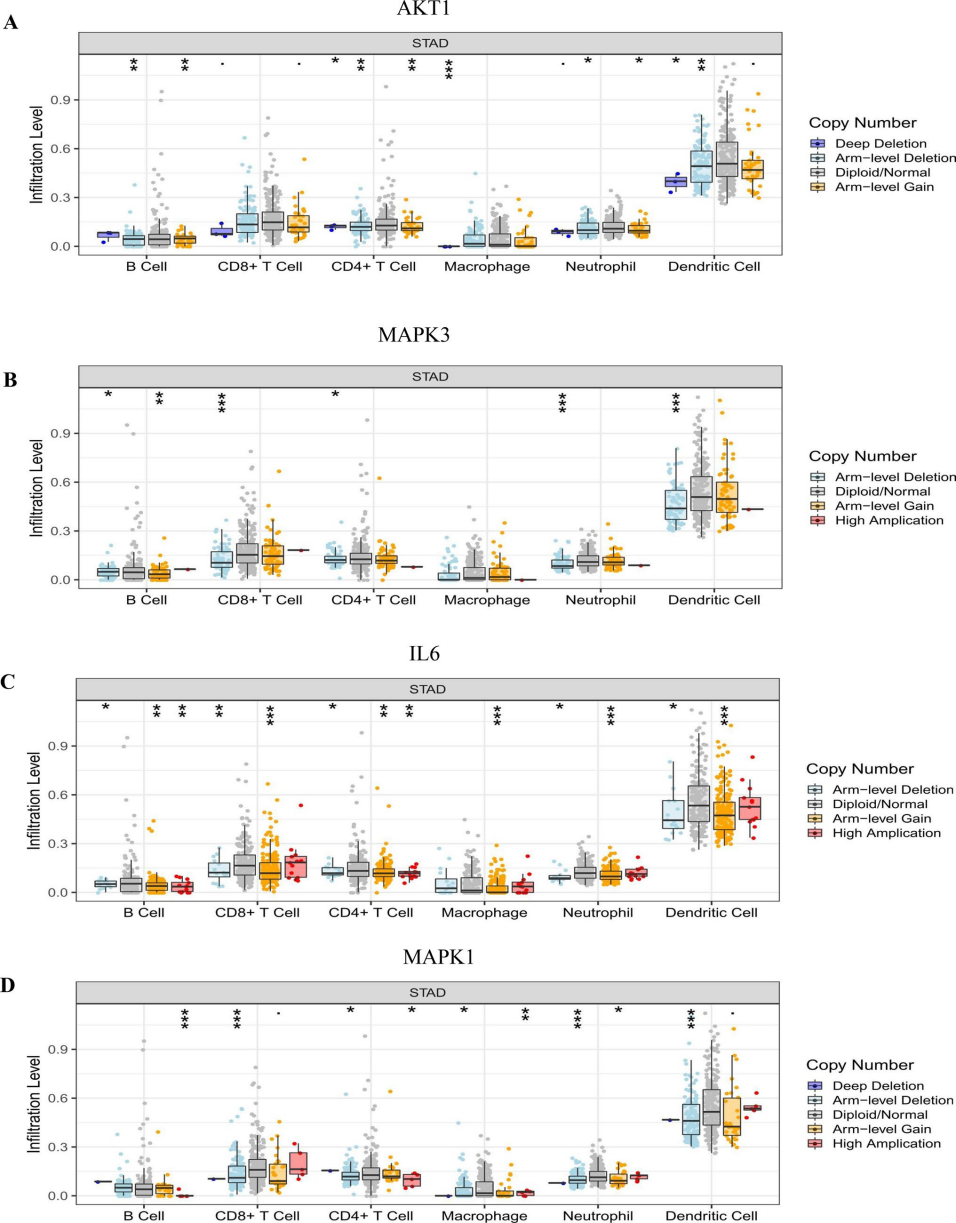

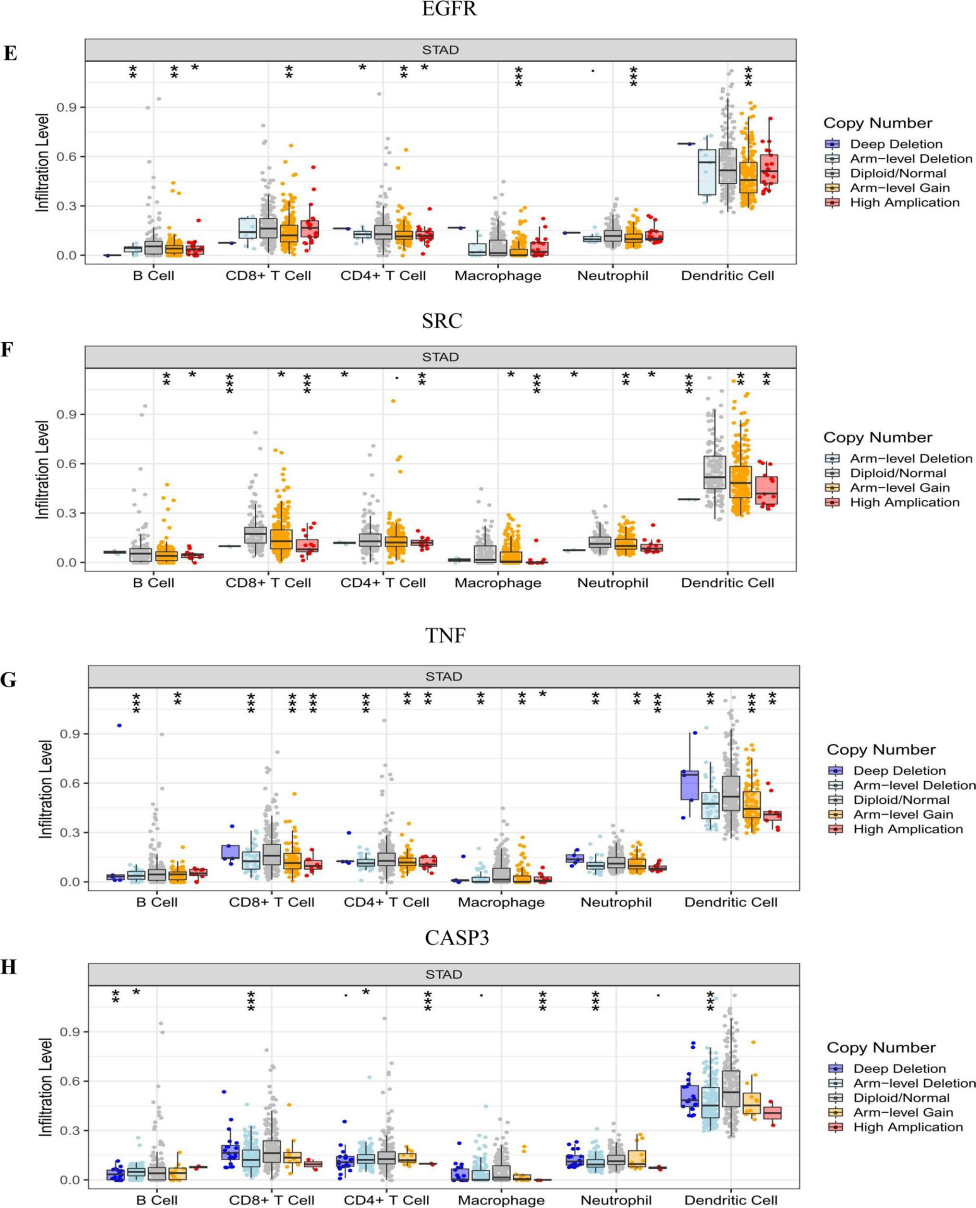

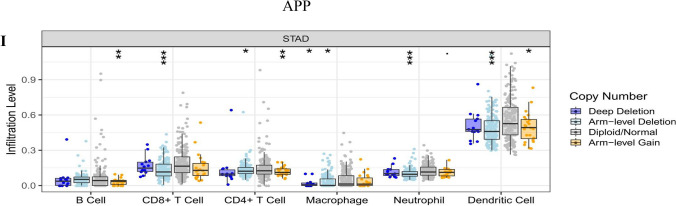
Immune cell infiltration of hub targets. Immune cell infiltration of (**A**) AKT1, (**B**) MAPK3, (**C**) IL6, (**D**) MAPK1, (**E**) EGFR, (**F**) SRC, (**G**) TNF, (**H**) CASP3, and (**I**) APP in the TIMER database.

**Figure 14:**
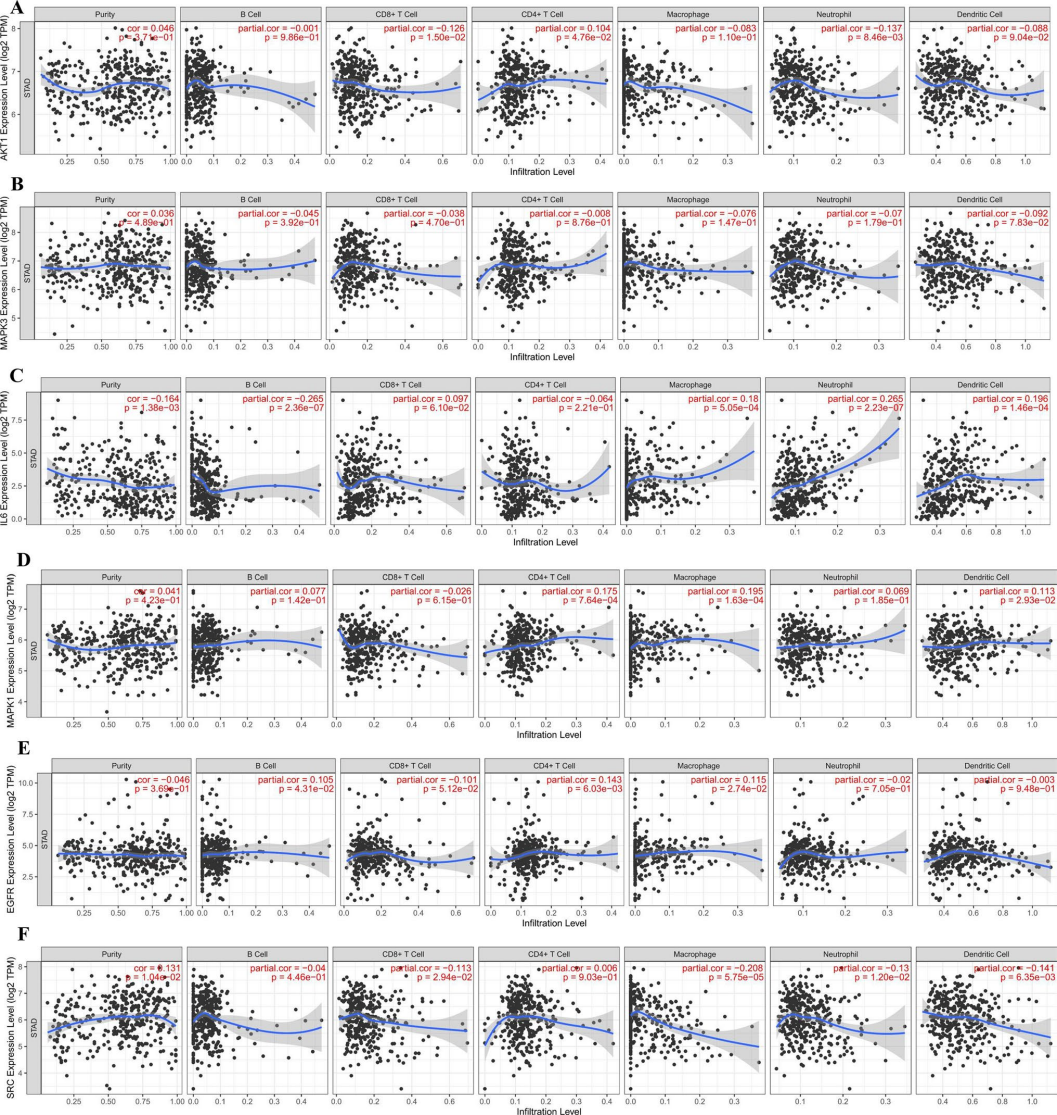

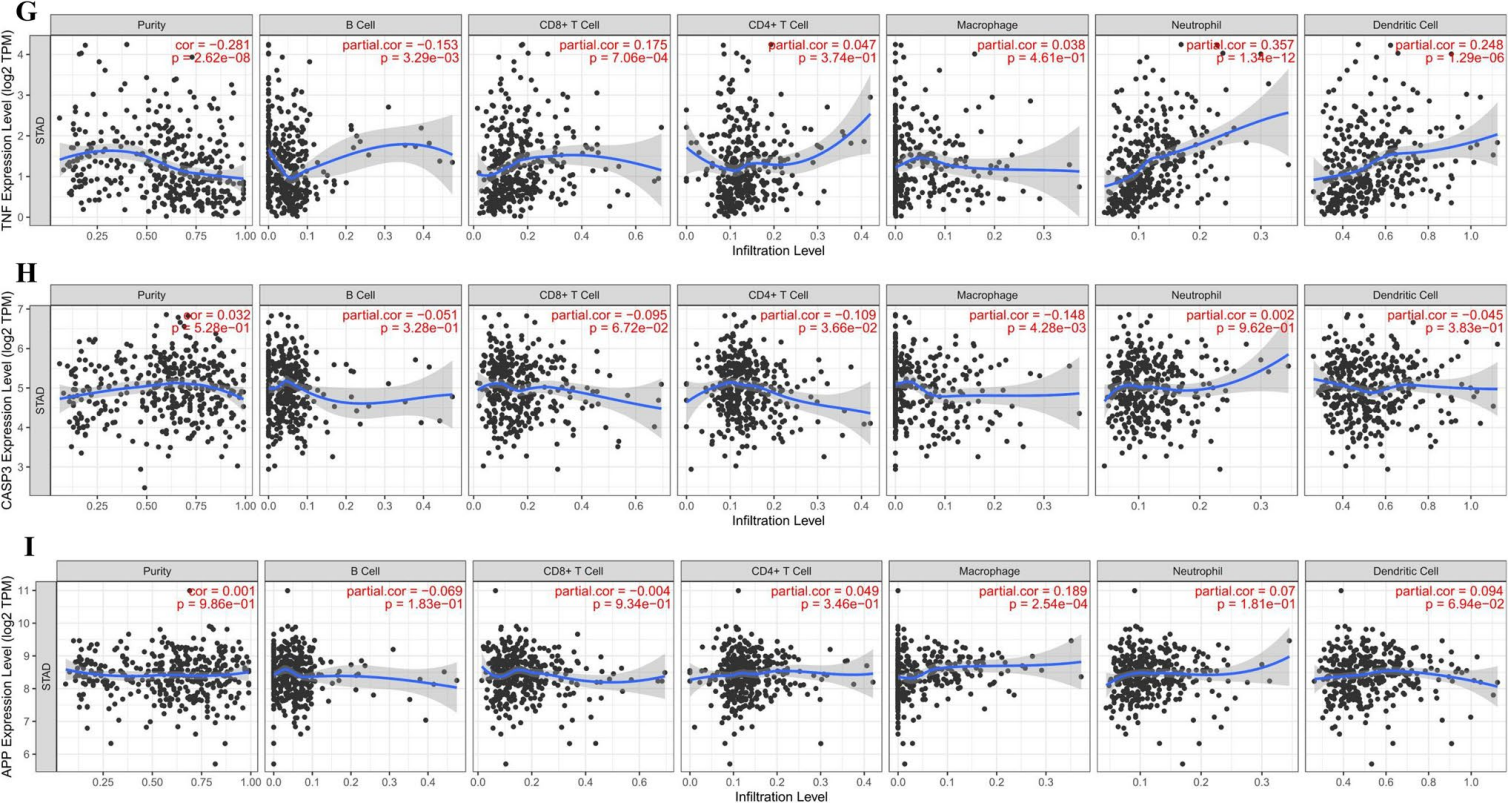
Correlation analysis of hub targets and immune cell infiltration. Correlation analysis of immune cell infiltration and (**A**) AKT1, (**B)** MAPK3, (**C**) IL6, (**D**) MAPK1, (**E**) EGFR, (**F**) SRC, (**G**) TNF, (**H**) CASP3, and (**I**) APP in the TIMER database.

The CNA of the hub target signature significantly affected the fraction of B cells, CD4+ T cells, CD8+ T cells, neutrophils, macrophages, and dendritic cells (DCs) in GC (Fig. 13). The association between hub target expression and the immune infiltrate is shown in Fig. 14. There is no information about CXCL8 in TIMER. Among them, the expression level of IL6 was significantly negatively correlated with B cells (cor =  − 0.256, P < 0.0001) and significantly positively correlated with neutrophils (cor = 0.265, P < 0.0001). SRC was significantly negatively correlated with macrophages (cor =  − 0.208, P < 0.0001). TNF was significantly negatively correlated with purity (cor =  − 0.281, P < 0.0001) and was significantly positively correlated with neutrophils and dendritic cells (cor = 0.357, P < 0.0001; cor = 0.248, P < 0.0001). The above results indicated that the hub targets play a critical role in regulating the tumor immune microenvironment of GC patients.

### Experimental verification in vitro

#### Eremanthin inhibited the proliferation of AGS cells

**Figure 15:**
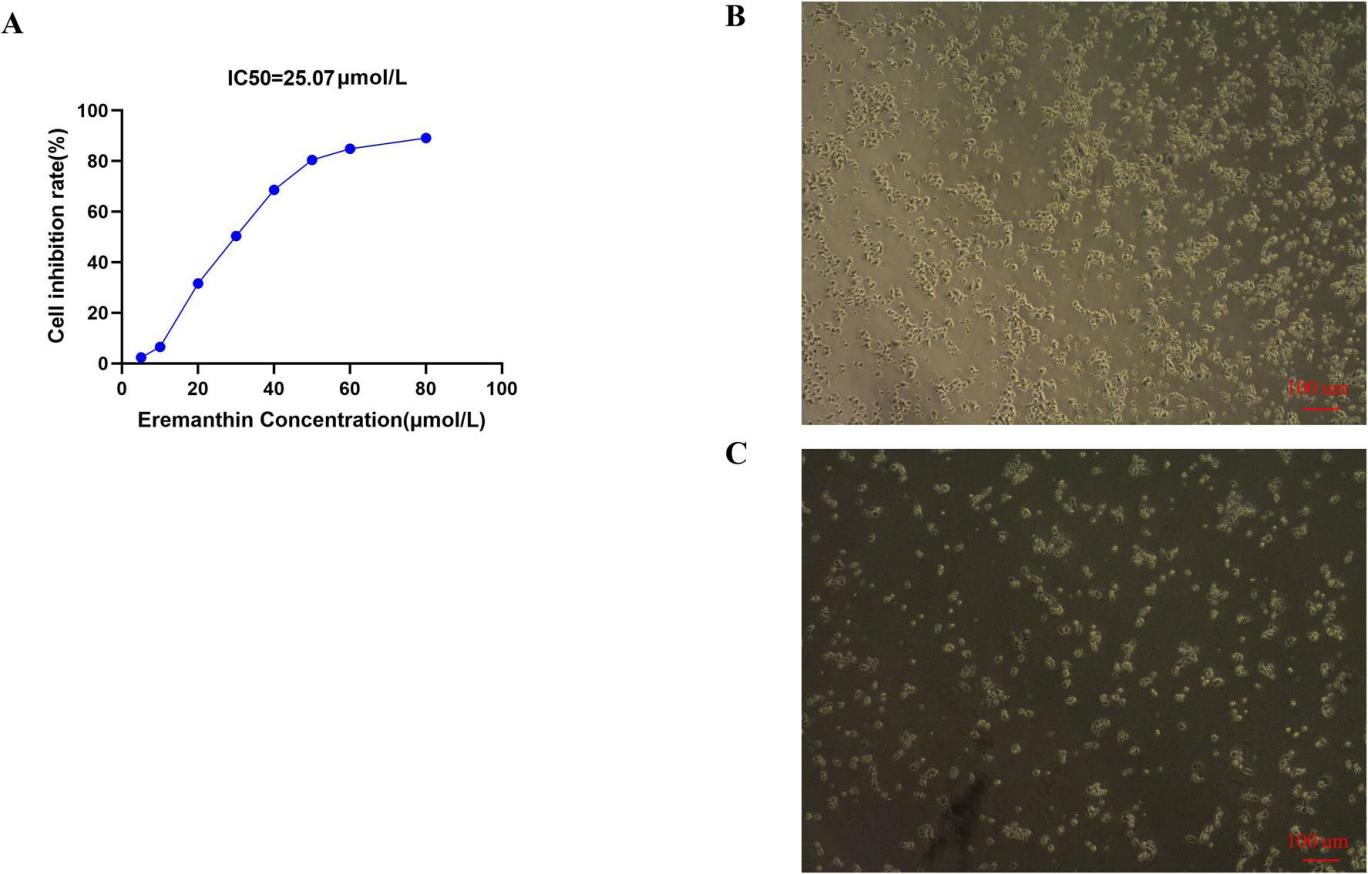
The graph of eremanthin inhibiting AGS growth. (**A–C**) show the growth state of AGS cells in different groups after 24 h of dosing. (**A**) The IC50 of eremanthin on AGS cells was 25.07 μmol/L, (**B**) represents the control group, and (**C**) represents the eremanthin group.

Eremanthin was observed to inhibit AGS cell proliferation in a concentration-dependent manner. The 24 h IC50 was 25.07 μmol/L, revealing that eremanthin for the AGS cell line showed a significant reduction in IC50 values over time. The cell viability curves are shown in Fig. 15. We chose this concentration for the follow-up experiment.

#### qRT–PCR

**Figure 16:**
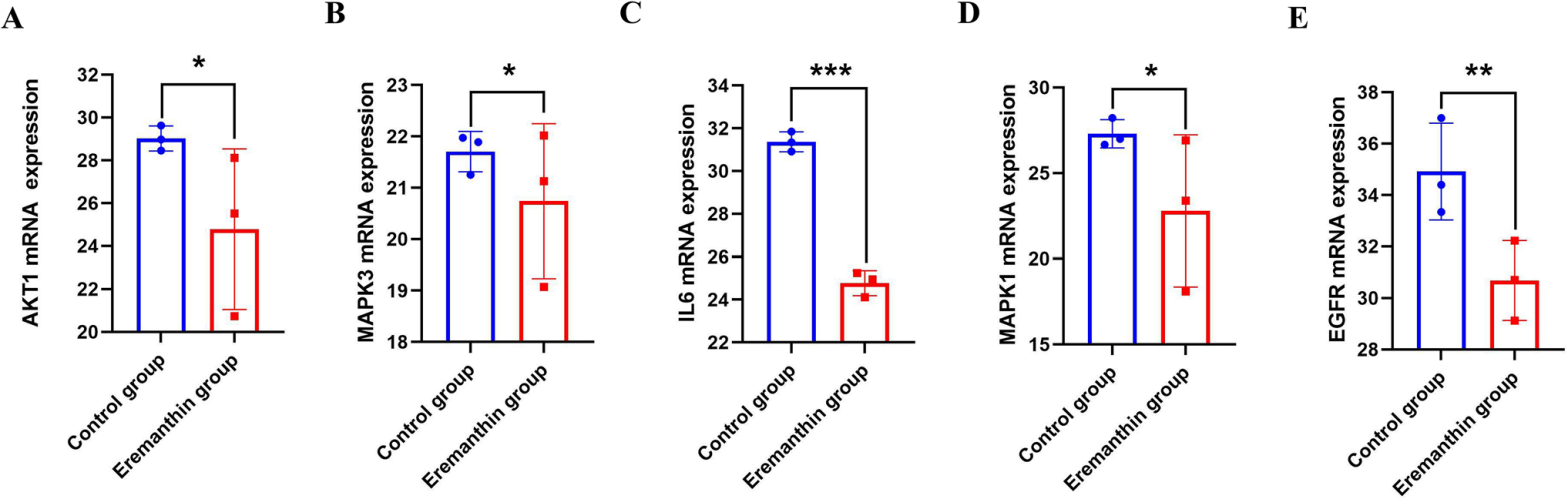
Effect of eremanthin on AKT1, MAPK3, IL6, MAPK1, and EGFR mRNA expression. (**A**) Relative mRNA expression of AKT1, (**B**) relative mRNA expression of MAPK3, (**C**) relative mRNA expression of IL6, (**D**) relative mRNA expression of MAPK1, and (**E**) relative mRNA expression of EGFR (*P < 0.05, **P < 0.01, ***P < 0.001).

Several hub targets, including AKT1, IL6, MAPK1, MAPK3, and EGFR mRNA expression levels were validated by PCR. Eremanthin was found to decrease the mRNA expression levels of AKT1, IL6, MAPK1, MAPK3, and EGFR. The results are depicted in Fig. 16. AKT1 is a key target of the PI3K-Akt signaling pathway. IL6 is one of the most important cytokine families in tumor occurrence and metastasis. The MAPK signaling pathway is a key pathway involved in GC. Target inhibition of EGFR expression can induce tumor cell death. The above experimental results proved that the main compound eremanthin was involved in the occurrence and development of GC.

## Discussion

It has been verified that TCM has the characteristics of “multiple components, multiple targets, and multiple pathways” in the treatment of GC^6^. According to the results achieved here, 106 compounds in AR-AF with 12,844 compound targets were identified, suggesting that AR-AF exerted its pharmacological effects on treating GC via multiple targets. Eremanthin, cynaropicrin, and aceteugenol were identified as the vital main compounds with the highest degrees. Eremanthin exerts anticancer activity on human cervical cancer cells by inhibiting the PI3K/AKT signaling pathway^47^. Studies have reported that cynaropicrin has a good antitumor effect and has a certain inhibitory effect on colorectal cancer^48^, melanoma^49^, and thyroid cancer^50^. Aceteugenol has a good antioxidant effect^51^. On the whole, it was speculated that AR-AF is a multicomponent herb pair with multitarget therapeutic effects. Associations between these active compounds and GC should be supposed to be deeply investigated.

GO and KEGG enrichment analyses showed that AR-AF regulates the proliferation, apoptosis, migration and angiogenesis of tumor cells by regulating signaling pathways, including the cAMP signaling pathway and PI3K-Akt signaling pathway, thereby playing a role in treating GC.

In this paper, AKT1, MAPK3, IL6, MAPK1, EGFR, SRC, TNF, CXCL8, CASP3, and APP were identified as hub targets. AKT is a serine/threonine kinase located downstream of PI3K. Activated AKT1 can induces the proliferation and activation of gastric cancer cells^52,53^. The positive rate of AKT1 expression in GC tissue is significantly higher than that in adjacent tissues, and it participates in the occurrence and development of GC^54,55^. MAPK3 is a serine/threonine protein kinase that can respond to a variety of extracellular stimuli. MAPK3 is related to tumor cell development, differentiation, apoptosis, angiogenesis, invasion and metastasis^56^. Mitogen-activated protein kinase 1 (MAPK1) has been confirmed as an essential oncogene in the progression of GC, and its level is elevated in GC tissues and cells, which can promote the proliferation, migration, and invasion of GC cells^57^. MAPK3 is a member of the MAPK family. MAPK is an important signal transmitter in cells that can participate in a variety of biological processes, such as cell proliferation, differentiation, and immune defense, by phosphorylating nuclear transcription factors and related enzymes^58^. IL6 is a pleomorphic cytokine involved in various biological processes, such as inflammation and tumor development^59,60^. Elevated IL-6 can stimulate the excessive activation of STAT3, including angiogenesis and tumor metastasis^61^. Activation of IL-6/STAT3 inhibits the transcription of miR-520f-3p, which amplifies the excessive activation of STAT3 by targeting GP130. Therefore, targeting this pathway may have the potential to treat GC^62^. EGFR is associated with gastric mucosal hyperplasia and poor prognosis of GC^63^. SRC is a serine/threonine kinase that plays an important role in the development of many solid tumors^64^. SRC is highly expressed in GC^65,66^. Activated SRC can promote tumor cell proliferation^67^. TNF-α is mainly secreted by macrophages and participates in inflammation and the immune response. Its effect on tumors is bidirectional; high concentrations of TNF-α can selectively destroy the tumor vascular system and produce specific T cells that have an antitumor effect, while low concentrations of TNF-α bind to the receptor, activate the NF-κB signaling pathway, promote cell proliferation, inhibit cell apoptosis, and promote tumor progression^68,69^. CXCL and CXCR are endogenous ligands of chemokines or members of the receptor family and are closely related to a variety of cancers^70^. Helicobacter pylori (HP) induces macrophages to release CXCL8, thereby promoting the occurrence and development of GC^71^. CASP3 is an apoptotic gene, and miR-524-5p participates in the development of GC by regulating CASP3, which may provide new prospects for the diagnosis and treatment of GC^72^. APP is the main target that we screened for the treatment of GC, but there is no clear antitumor effect report, which is worthy of further study. Molecular docking validated that vital active compounds and major targets showed good binding interactions. It is suggested that the AR-AF herb pair may act on the major targets to play a key role in the treatment of GC.

The results of immune infiltrates showed that the CNA of the hub target signature significantly affected the fraction of B cells, T cells, neutrophils, macrophages and DCs in GC, which suggested that the hub targets can be used as a potential predictor of gastric cancer immunotherapy.

The experimental results in this study confirmed that the active compounds of AR-AF inhibited cell proliferation and induced apoptosis of AGS cells, thus delaying the progression of GC, which will provide a preliminary basis for future clinical trials.

## Conclusion

In this study, eremanthin, cynaropicrin, and aceteugenol were identified as the vital active compounds, and AKT1, MAPK3, IL6, MAPK1, as well as EGFR were considered as the major targets. Molecular docking revealed that these active compounds and major targets showed good binding interactions. AR-AF regulates the proliferation, apoptosis, migration and angiogenesis of tumor cells by regulating THE cAMP signaling pathway, and PI3K-Akt signaling pathway. Experimental studies provided evidence that AR-AF showed therapeutic effects on GC by regulating related target proteins, inhibiting cell proliferation, and increasing the cell apoptosis rate. This study demonstrated potential pharmacological mechanisms of AR-AF acting on GC, and it can be referenced for the clinical application of AR-AF.

## Supplementary Information

Supplementary Table S1.

Supplementary Table S2.

Supplementary Table S3.

## Data Availability

The data used to support the findings of this study are included within the article.

## Abbreviations

GC: Gastric cancer
AR: Aucklandiae Radix
AF: Amomi Fructus
TCMSP: Traditional Chinese Medicine Systems Pharmacology Database and Analysis Platform
TCM: Traditional Chinese medicine
OB: Oral bioavailability
DL: Drug-likeness
GO: Gene Ontology
BP: Biological process
CC: Cellular component
MF: Molecular function
FDR: Error detection rate
KEGG: Kyoto Encyclopedia of Genes and Genomes
PPI: Protein–protein interaction
OS: Overall survival
IHC: Immunohistochemical
